# Global mapping of RNA N^6^-methyladenosine (m^6^A) in human subcutaneous and visceral adipose tissue reveals novel targets that correlate with clinical variables of obesity

**DOI:** 10.1186/s40364-025-00857-0

**Published:** 2025-11-12

**Authors:** Torunn Rønningen, Yong Zeng, Mai Britt Dahl, Junbai Wang, Tina Visnovska, Tone Møller Tannæs, Lars la Cour Poulsen, Akin Cayir, Stina Ingrid Alice Svensson, Marius Svanevik, Jens Kristoffer Hertel, Jøran Hjelmesæth, Jon A. Kristinsson, Tom Mala, Matthias Blüher, Housheng Hansen He, Tone Gretland Valderhaug, Yvonne Böttcher

**Affiliations:** 1 EpiGen, Medical Division, Akershus University Hospital, Postboks 1000, Lørenskog, 1478 Norway; 2 Department of Clinical Molecular Biology, EpiGen, Institute of Clinical Medicine, University of Oslo, Oslo, Norway; 3 Princess Margaret Cancer Center, University Health Network, Toronto, Ontario Canada; 4 Bioinformatics Core Facility, Oslo University Hospital, Oslo, Norway; 5 Department of Endocrinology, Obesity and Nutrition, Vestfold Hospital Trust, Tønsberg, Norway; 6 Department of Endocrinology, Morbid Obesity and Preventive Medicine, Institute of Clinical Medicine, University of Oslo, Oslo, Norway; 7 Department of Endocrinology, Morbid Obesity and Preventive Medicine, Oslo University Hospital, Oslo, Norway; 8 Department of Gastrointestinal and Children Surgery, Institute of Clinical Medicine, University of Oslo, Oslo, Norway; 9 Helmholtz Institute for Metabolic, Obesity and Vascular Research (HI-MAG) of the Helmholtz Zentrum München at the University of Leipzig and University Hospital, Leipzig, Germany; 10 Department of Endocrinology, Akershus University Hospital, Lørenskog, Norway

**Keywords:** Obesity, Fat distribution, Adipose tissue, N^6^-methyladenosine, Epitranscriptomics

## Abstract

**Background:**

Obesity is a major health challenge and fat accumulation in visceral depots is more strongly associated with metabolic comorbidities than deposition in subcutaneous depots. Epitranscriptomic regulation of gene expression by N^6^-methyladenosine (m^6^A) influences various aspects of RNA metabolism, however the m^6^A methylome in human adipose tissue and its relationship with fat distribution has not yet been investigated in detail.

**Methods:**

In this study, we performed epitranscriptomic mapping of m^6^A in intra-individually paired samples of subcutaneous (SAT) and omental visceral adipose tissue (OVAT) from women with normal weight (BMI ≤25, *n* = 3) and obesity (BMI ≥35, *n* = 10) using meRIP-seq (discovery cohort). We further investigated differential m^6^A methylation for specific target genes in a larger cohort of individuals with obesity (*n* = 72, validation cohort) using meRIP-qPCR. meRIP-seq was performed for primary adipocytes from a subset of the patients (*n* = 4) to account for cell type specific differences.

**Results:**

We here provide the first global map of m^6^A in human adipose tissue in paired samples of SAT and OVAT. We show an overall high overlap in m^6^A sites between individuals and depots, but also distinct depot-specific differences. We identify 339 target genes showing depot-specific m^6^A methylation. Depot-specific methylation was validated for selected sites in *SEMA3A*, *SNAP47* and *PPP1R9A* in a larger validation cohort. We additionally identify differentially methylated targets between lean individuals and individuals with obesity, including *TSC22D1*, *FMNL2* and *IL1R1*. By combining data from primary adipocytes with data from corresponding bulk adipose tissue, we identified a higher number of genes containing m^6^A in non-adipocyte cells in OVAT compared to SAT. Mechanistically, we show for selected targets that m^6^A affects RNA lifetime in pre-adipocyte cell culture models. Importantly, m^6^A methylation in selected targets correlates with clinically important variables related to obesity, fat distribution and glucose metabolism.

**Conclusions:**

We identify a catalogue of novel targets showing adipose tissue depot specific m^6^A methylation, with potential as biomarkers in metabolic disease. Our findings underscore the regulatory role of m^6^A in obesity and provide valuable insights for future research. The datasets generated represent a significant resource for further insight in adipose tissue biology and its implications for metabolic health.

**Supplementary Information:**

The online version contains supplementary material available at 10.1186/s40364-025-00857-0.

## Background

White adipose tissue plays a central role in development of obesity and associated co-morbidities [1]. Adipose tissue distribution is an important risk factor for developing metabolic dysfunction, in particular, accumulation of fat in visceral depots is closely linked to metabolic dysfunction and obesity-related comorbidities [2]. Despite advancements in our understanding of obesity and adipose tissue biology revealing distinct gene expression and epigenetic profiles, which differentially correlate with clinical variables related to obesity [3–6], the underlying mechanisms for the association between anatomic fat storage and metabolic dysfunction are not completely understood.

In addition to epigenetic regulation, post-transcriptional RNA modifications, known as epitranscriptomics serves as a crucial mechanism for fine tuning gene expression. Among the RNA modifications, N^6^-methyladenosine (m^6^A) is the most abundant in eukaryotic mRNA and plays a pivotal role in various aspects of RNA metabolism including RNA stability, splicing, nuclear transport and translational efficiency [7]. m^6^A is dynamically regulated by the m^6^A “writer” complex, including the core components METTL3, METTL14 and WTAP, as well as the “eraser” demethylases ALKBH5 and FTO [8]. In addition, several m^6^A “reader” proteins have been identified, which either recognize m^6^A directly or indirectly through binding to m^6^A induced secondary structures on RNA [9]. The functional consequences of m^6^A methylation depend on the binding of associated reader proteins exerting diverse effects on RNA metabolism and translation [10]. Genetic variants in the Fat mass and obesity associated (*FTO)* gene, encoding a m^6^A demethylase, have been consistently associated with body mass index (BMI) across multiple populations [11, 12], suggesting a potential association between m^6^A and obesity. More specifically, *FTO* influences adipogenesis by regulating *JAK2* expression, *STAT3* phosphorylation, and C/*EBPβ* transcription [13] and affects the splicing of the *RUNX1T1* gene and alters its m^6^A levels [14].

Importantly, numerous studies suggest a role of m^6^A in disease development, with recent evidence highlighting its involvement in adipogenesis and fat metabolism [15, 16]. We have recently shown that the expression of several m^6^A regulators are adipose tissue depot- and obesity-specific, whilst gene expression levels significantly correlate with clinical variables of obesity [17]. Recent findings revealed m^6^A’s role in regulating the release of free fatty acids from adipose tissue in response to hypoxia, particularly through its influence on the RNA decay of monoglyceride lipase [18], Additionally, METTL3 was recently shown to play an important role in regulation of beiging of white adipose tissue and metabolic regulation in mice [19] highlighting the regulatory significance of m^6^A in adipose tissue metabolism. Transcriptome wide mapping of m^6^A in adipose tissue from other vertebrates such as pigs and chickens shows that transcripts marked by m^6^A are involved in regulation of fat metabolism and deposition [20, 21]. However, similar data in human tissue remain limited. To our knowledge, the only available transcriptome-wide m^6^A data from human adipose tissue comes from a comparative study on m^6^A across various human tissues. This study includes one sample from *post mortem* subcutaneous femoral adipose tissue [22]. So far, there is a knowledge gap in understanding whether m^6^A methylation confers depot-specific signatures in adipose tissue, whether it affects RNA metabolism regulating gene expression and to what extent this correlates with clinical variables related to obesity.

In this study, we hypothesised that adipose tissue contains distinct depot-specific m^6^A signatures, which associate with obesity. We here employed meRIP-seq on intra-individually paired biopsies from abdominal subcutaneous (SAT) and omental visceral adipose tissue depots (OVAT) from individuals with obesity (*n* = 10) as well as normal-weight controls (*n* = 3). We identified depot-specific as well as obesity-specific m^6^A signatures. We further validated m^6^A levels at identified targets in a validation cohort of paired samples of SAT and OVAT from patients with obesity (*n* = 72). Furthermore, we combined data from whole adipose tissue biopsies with data from primary adipocytes to decipher cell type-specific contribution to m^6^A signatures. Finally, we investigated whether m^6^A levels correlate with clinically important variables related to obesity, fat distribution and glucose metabolism.

## Methods

### Collection of human adipose tissue biopsies

Adipose tissue (AT) biopsies were obtained from abdominal subcutaneous (SAT) and omental visceral depots (OVAT) during bariatric surgery (individuals with obesity, BMI ≥35 kg/m^2^) or during cholecystectomy (normal weight, BMI < 25 kg/m^2^). The discovery cohort comprised 10 individuals with obesity (marked A) and 4 normal weight individuals (marked K), while the validation cohort comprised 72 patients with obesity. Biopsies were immediately snap frozen on dry ice/liquid nitrogen to prevent degradation and stored at −80C until further processing. Clinical and anthropometric traits of the patients including waist and hip circumference, waist to hip ratio (WHR), Body mass index (BMI), Fasting serum glucose, fasting serum insulin, triglycerides, total cholesterol, HDL cholesterol, LDL cholesterol, HbA1c and HOMA-IR were measured at baseline. The summarized clinical characteristics of the patient cohorts are shown in Table 1. All study protocols have been approved by the Regional Ethics Committee in Norway (2018/1399; 2013/2042). All participants gave written informed consent before taking part in the study.

**Table 1:** Clinical characteristics of the study participants. Results are shown as mean ± SD. P-values were calculated using independent samples t-test

	Discovery cohort			Validation cohort
Clinical parameters at baseline	Obese	Lean	*P*-value	Obese
N	11	4		72
Age, years	43.6 ± 9.5	43.3 ± 12.1	0.949	43.0 ± 11.5
Male/female	0/11	0/4		25/47
T2D yes/no	3/7	0/4		14/58
BMI (kg/m^2^)	44.0 ± 6.5	24.6 ± 1.9	**5.79E-05**	45.1 ± 6.8
Waist circumference (cm)	116.9 ± 10.8	81 ± 5.35	**2.93E-05**	125.5 ± 15.3
Hip circumference (cm)	131.9 ± 14.8			132.4 ± 12.7
Waist-to-hip ratio (WHR)	0.89 ± 0.09			0.95 ± 0.12
Total cholesterol (mmol/L)	4.62 ± 0.09	4.73 ± 1.60	0.881	4.86 ± 0.92
HDL cholesterol (mmol/L)	1.34 ± 0.33	1.5 ± 0.36	0.419	1.37 ± 1.31
LDL cholesterol (mmol/L)	2.60 ± 0.75	3.20 ± 1.30	0.317	2.89 ± 0.82
Triglycerides (TG, mmol/L)	1.60 ± 0.64	0.88 ± 0.28	0.052	1.85 ± 0.80
Fasting serum glucose (FSG, mmol/L)	6.85 ± 2.19	5.25 ± 0.47	0.041	6.37 ± 2.51
Fasting serum insulin (FSI, pmol/L)	143.8 ± 119.0			220.4 ± 252.6
HOMA IR (mmol/l*pmol/L/135)	7.70 ± 8.03			10.47 ± 12.34
HbA1c (mmol/mol, IFCC)	46.05 ± 15.23	36.8 ± 0.50	0.07	42.33 ± 10.73

### Adipocyte isolation

Primary adipocytes (AC) were isolated from fresh adipose tissue biopsies from a subgroup of the patients (*n* = 5). Briefly, tissue was rinsed, minced in smaller pieces and incubated with 2 mg/ml collagenase type I (Worthington) in AIS buffer (5.5 mM D-glucose, 4% BSA, 0.8 mM ZnCl_2_, 500 nm adenosine in Krebs-Ringer-bicarbonate-HEPES, pH 7.2) at 37 °C for 45 min. Digested tissue was filtered through 400 µm nylon mesh and centrifuged at 150 g for 8 min to separate adipocytes from the stromal vascular fraction (SVF). The floating adipocyte fraction was washed twice in AIS buffer, snap-frozen in liquid N_2_ and stored at −80 °C before further processing.

### Sample inclusion

For the discovery cohort, RNA-seq analyses were performed for all adipose tissue (*n* = 10/4) and adipocyte samples (*n* = 5). Due to insufficient RNA yield, K5-SAT and K5-OVAT were excluded from meRIP experiments. Additionally, meRIP-seq results for A17-OVAT-AC did not pass quality control for m^6^A peak calling (metagene plot, Fig. S2B) and this and the corresponding SAT-AC sample were excluded from further m^6^A analyses. The resulting m^6^A-seq dataset contains paired data from SAT and OVAT from 10 patients with obesity and 3 normal weight individuals, as well as paired data from ACs from SAT and OVAT from 4 patients with obesity. For the validation cohort, two samples showed high signal to noise ratio and were excluded from further analysis, resulting in a dataset with paired samples from 72 patients.

### Cell culture and transfections

Pre-adipocytes originating from OVAT and SAT (#OP-F-3 and #SP-F-3; ZenBio) were from non-smoking, non-diabetic Caucasian women with obesity matched for BMI (BMI 35). Cells were cultured in DMEM/F12 (Gibco) with 10% Fetal bovine serum (FBS), 1% Penicillin/Streptomycin and 1 ng/ml basic fibroblast growth factor (bFGF). For siRNA knockdown experiments, cells were transfected with RNAiMax transfection reagent (Thermo Fisher Scientific) using a forward transfection protocol with 10 nM siRNA targeting *METTL3* (siMETTL3, #s32142 ThermoFisher Scientific) or nontargeting control siRNA (siCtrl, #4390843 ThermoFisher Scientific).

### RNA stability assay

RNA stability of selected target genes was determined using Click-iT™ Nascent RNA Capture Kit according to protocol (#C10365, Thermo Fisher Scientific). Briefly, pre-adipocytes were transfected with siRNA targeting *METTL3* (#s32142, Thermo Fisher Scientific) or a non-specific siRNA control (#4390844, Thermo Fisher Scientific). 72 hours after transfection, cells were incubated with 0.2 mM 5-ethynyl uridine (EU) for 24 h. Following incubation, cells were washed twice with PBS and replaced with fresh medium. RNA was then extracted after 0, 2, 4, and 8 h. 5 ug RNA was used as input for the ClickIT biotinylation reaction. Biotinylated RNA was pulled down with streptavidin beads, and 500 ng RNA was used as input for reverse transcription using SuperScript™ VILO™ cDNA Synthesis Kit (Thermo Fisher Scientific). The relative amount of biotinylated transcripts were quantified by qPCR on a QuantStudio Flex 7 system (Thermo Fisher Scientific) using PowerUP SYBR green mastermix (Thermo Fisher Scientific) with *GAPDH* and *ACTB* as reference genes. Primer sequences used for RT-qPCR are shown in Table S1.

### Western blot

For protein analyses, cells pellets were lysed in RIPA buffer and sonicated for 30 seconds. 10–20 µg protein/well was separated by SDS-PAGE and transferred onto PVDF membranes. Membranes were blocked with non-fat dry milk, and incubated with primary antibodies (α-METTL3, #15073–1-AP, Proteintech; GAPDH, #2118, Cell signaling technologies) at 4 °C over night. Blots were incubated with Goat anti-rabbit IgG-HRP antibodies (#4030–05, Southern Biotech) for 1 hour at room temperature before incubation with ECL prime western blotting detection reagent (#RPN2236, Amersham). Images were acquired using a LAS 3000 mini imager system (Fujifilm). Quantification of protein levels was performed using MultiGauge software (Fujifilm).

### RNA isolation

For cultured pre-adipocytes, RNA was extracted with the RNeasy mini kit (Qiagen) using the standard protocol. Primary adipocytes and adipose tissue were lysed in Qiazol and homogenized passing the lysate through a 18 G needle (adipocytes) or with ceramic beads in a MP BIO Fastprep^TM^ 5 G unit with 6.0 m/s for 2 × 30 sec (tissue). The homogenized lysate was centrifuged for 5 minutes and the floating lipid phase was discarded. Phase separation was performed with chloroform. RNA from the aqueous phase was precipitated with 70% EtOH, purified on RNeasy MinElute spin columns and subjected to on-column DNase digestion using the RNeasy Micro Kit (Qiagen). RNA integrity (RIN) was evaluated on Bioanalyzer (Agilent). Samples with RIN < 5 were excluded from further analyses.

### Genotyping

Genomic DNA was extracted from OVAT using the GenElute Mammalian Genomic DNA Miniprep kit (Sigma-Aldrich,) according to protocol. 20 ng DNA was used as input for genotyping with Taqman SNP Genoyping assays (#4351379, Thermo Fisher Scientific) using probes against rs9939609 (Assay ID:C__30090620_10, Thermo Fisher Scientific). Samples were run in duplicates on the QuantStudio 6 Pro System (Thermo Fisher Scientific) following manufacturers recommendations and analysed using the Design and Analysis 2.4.0 Software (Thermo Fisher Scientific).

### m^6^A dot blot

mRNA was extracted from total RNA with Dynabeads Oligo(dT)_25_ (Thermo Fisher Scientific) and applied to Hybond+ membrane (Amersham) using a Bio-Dot Apparatus (Bio-Rad) followed by UV crosslinking for two cycles of 1,200 µJ [x100] with a UVP Crosslinker. Membranes were blocked with 5% non-fat dry milk and incubated with α-m^6^A primary antibody (#202 003, Synaptic Systems). Blots were incubated with Goat anti-rabbit IgG-HRP antibodies (#4030–05, Southern Biotech) for 1 hour, incubated with ECL reagent (#RPN2236, Amersham) and images were acquired on a LAS3000 mini imager system (Fujifilm). Membranes were stained with methylene blue (0.02% methylene blue in 0.3 M NaOAc (pH 5.5)) as RNA loading control. Quantification of protein levels was performed using MultiGauge software (Fujifilm).

### Immunoprecipitation of N^6^-methylated RNA (meRIP)

m^6^A immunoprecipitation experiments were performed as previously described [23] with minor modifications. Briefly, 3–5 µg total RNA was chemically fragmented into 200 nucleotide fragments (#AM8740, Thermo Fischer Scientific). Methylated RNA fragments were immunoprecipitated (IP) using m^6^A targeting antibodies (#ABE572, Millipore, discovery cohort; ab286164, Abcam, validation cohort) coupled to protein A and G Dynabeads (1:1 ratio, Thermo Fisher Scientific) for 2 hours. E.Coli K12 RNA was included as spike-in control. Following IP, magnetic beads were washed twice in 1 ml IP buffer (150 mM NaCl, 10 mM Tris-HCl [pH 7.5], 0.1% IGEPAL CA-630 in nuclease-free H_2_O), twice in 1 ml low-salt IP buffer (50 mM NaCl, 10 mM Tris-HCl [pH 7.5], 0.1% IGEPAL CA-630 in nuclease-free H_2_O), and twice in 1 ml high-salt IP buffer (500 mM NaCl, 10 mM Tris-HCl [pH 7.5], 0.1% IGEPAL CA-630 in nuclease-free H_2_O) at 4 °C. RNA enriched with m^6^A was eluted from the beads with RLT buffer (Qiagen; Germany) and purified on MinElute spin columns (Qiagen). IP RNA was eluted in 14 ul nuclease free-water.

### meRIP qPCR

For meRIP-qPCR in the validation cohort, IPs were performed on 4 µg total RNA in 3 separate batches, paired SAT and OVAT were always run in the same batch. Calibrator samples were included to account for batch variation. 5 µl IP RNA and 400 ng input RNA was subjected to cDNA synthesis using the High Capacity Reverse Transcription Kit (ThermoFisher Scientific). qPCR was performed on a QuantStudio Flex 7 system (ThermoFisher Scientific) with PowerUP SYBR green mastermix (ThermoFisher Scientific) using specific primers towards m^6^A DMRs and *GAPDH* as negative control (Table S1). Targets from the top 50 DMRs (Table 2) with TPM ≥10 (from RNAseq) for both SAT and OVAT were chosen for validation.

**Table 2:** Top 50 differentially methylated regions between SAT and OVAT identified by RADAR ranked by p-value. The complete list of differentially methylated regions are in table S6 (*n* = 13, log2FC > 0.5, fdr < 0.1). ncRNA = non coding RNA; UTR = untranslated region; CDS = coding sequence. Positive log2FC - hypermethylated in OVAT; negative log2FC – hypermethylated in SAT

Gene_name	Position	Log2 Fold Change	P-value	Genomic feature
*IGHG1*	chr14:105737230-105737428(-)	**5,991465**	**0**	3‘UTR
*IGHG1*	chr14:105736540-105736935(-)	**4,877738**	**0**	3‘UTR
*RP1-27O5.3*	chr1:32485318-32536197 (+)	**4,532599**	**0**	intron
*NKX2-3*	chr10:99535926-99536125 (+)	**4,276666**	**0**	3‘UTR
*HNRNPA1P57*	chr2:41156675-41156774 (+)	**3,925268**	**0**	pseudogene
*PRR15L*	chr17:47952440-47952539 (-)	**3,893389**	**0**	3‘UTR
*RP13-895J2.4*	chr12:132279739-132279838 (-)	**3,764296**	**0**	ncRNA
*HS3ST6*	chr16:1911561-1911855 (-)	**3,740048**	**0**	stop codon
*LRRC38*	chr1:13475552-13475651 (-)	**3,713572**	**0**	3‘UTR
*PRR15L*	chr17:47953008-47953201 (-)	**3,710519**	**0**	CDS
*C1QL4*	chr12:49336990-49337089 (-)	**3,326234**	**0**	5‘UTR
*DSC3*	chr18:30996855-30996954 (-)	**3,295837**	**0**	CDS
*CDH1*	chr16:68833457-68833556 (+)	**3,183249**	**0**	stop codon
*AP002884.2*	chr11:112179319-112193607 (+)	**3,068053**	**0**	CDS
*CHST4*	chr16:71537264-71537461 (+)	**3,044522**	**0**	CDS
*FLRT3*	chr20:14323886-14323985 (-)	**3,039217**	**0**	3‘UTR
*HSD17B2*	chr16:82098236-82098335 (+)	**2,901422**	**0**	CDS
*RMI2*	chr16:11316853-11316952 (+)	**2,811591**	**0**	CDS/intron
*CHGB*	chr20:5923701-5923800 (+)	**2,74084**	**0**	CDS
*EPPK1*	chr8:143870841-143870940 (-)	**2,639057**	**0**	CDS
*RP11-707A18.1*	chr4:64915873-64915972 (-)	**2,572612**	**0**	ncRNA
*SYT9*	chr11:7466876-7466975 (+)	**2,550865**	**0**	3‘UTR
*NEU4*	chr2:241808925-241809024 (+)	**2,461917**	**0**	3‘UTR
*LINC00842*	chr10:46398493-46398792 (+)	**-1,90248**	**7,89E-31**	ncRNA
*HAND2–AS1*	chr4:173527965-173528658 (+)	**-1,12008**	**1,30E-29**	ncRNA
*SEMA3A*	chr7:84194552-84194851 (-)	**2,225215**	**2,88E-21**	Start codon
*FAM101A*	chr12:124315330-124315627 (+)	**3,125088**	**3,94E-20**	3‘UTR
*MMP24–AS1*	chr20:35224579-35225620 (-)	**1,11177**	**4,04E-19**	intron
*DST*	chr6:56540823-56541121 (-)	**0,609262**	**2,19E-18**	CDS
*SNAP47*	chr1:227732576-227732775 (+)	**1,507599**	**5,52E-18**	5‘UTR
*DST*	chr6:56541222-56541521 (-)	**0,647192**	**6,19E-18**	CDS
*RP4-625H18.2*	chr6:19804752-19839080 (-)	**-1,45078**	**6,66E-17**	intron
*GATA5*	chr20:62464196-62464394 (-)	**4,469315**	**1,06E-16**	3‘UTR
*FAM101A*	chr12:124314438-124314735 (+)	**2,720669**	**2,90E-16**	Stop codon
*ANXA8L1*	chr10:46391517-46391616 (+)	**3,208152**	**3,33E-16**	3‘UTR
*LOXL2*	chr8:23404124-23425230 (-)	**1,291804**	**8,73E-16**	5‘UTR
*ARHGAP6*	chrX:11665203-11665601 (-)	**1,332344**	**3,73E-14**	5‘UTR
*NKX2-3*	chr10:99535726-99535825 (+)	**2,618438**	**5,31E-14**	3‘UTR
*CPA4*	chr7:130322597-130322696 (+)	**2,110213**	**1,26E-12**	Stop codon
*PPP1R9A*	chr7:94911116-94911414 (+)	**0,635517**	**1,44E-12**	CDS
*CASP8*	chr2:201240390-201240687 (+)	**1,3064**	**1,54E-12**	intron
*SNAP47*	chr1:227734171-227735113 (+)	**1,531476**	**1,81E-12**	5‘UTR
*CACNG4*	chr17:67031808-67032006 (+)	**3,321645**	**2,39E-12**	3‘UTR
*PKHD1L1*	chr8:109464488-109464786 (+)	**2,691463**	**6,59E-12**	CDS
*LOC728392*	chr17:5499626-5499824 (-)	**0,627783**	**7,86E-12**	ncRNA
*COL18A1*	chr21:45455785-45456082 (+)	**0,558065**	**9,32E-12**	CDS
*SEMA3B*	chr3:50276651-50276850 (+)	**0,704212**	**2,65E-11**	stop codon
*MIR100HG*	chr11:122101503-122101702 (-)	**-0,79942**	**3,05E-11**	ncRNA
*COL18A1*	chr21:45455586-45455685 (+)	**0,96501**	**2,58E-10**	CDS
*DLG2*	chr11:85626659-85627422 (-)	**-0,97117**	**3,86E-10**	5‘UTR

### Next generation sequencing

m^6^A-seq and RNA-seq libraries were generated using SMARTer® Stranded Total RNA-Seq Kit v2 - Pico Input (#634412, Takara) according to the protocol for fragmented RNA. 3,5 µl IP RNA and 50 ng input RNA were used for library construction. IP libraries were PCR amplified for 16 cycles, while 12 cycles were used for input RNA libraries. Library fragment size distribution was analysed with Bioanalyzer HS DNA kit (Agilent). Libraries were sequenced as paired ends with 150 cycles, on the HiSeq4000 platform (Illumina, San Diego, CA, USA) at the Norwegian Sequencing Centre, Oslo University Hospital.

### m^6^A-sequencing data analysis

Basic m^6^A-seq analyses were performed using an established pipeline as previously described [23]. Briefly, adapter sequences were trimmed from raw sequences using Cutadapt v.1.18, and sequencing reads were aligned to HG38 as single ends using STAR v.2.7.0 [24] with reference annotation GENCODE v.25. *E*. *coli* K-12 spike-in sequences were incorporated with the human genome for mapping. Uniquely mapped reads were selected by samtools v. 1.3.1 and were separated by strand with RSeQC v.2.6.1. All reads were extended to a length of 200 nt in the 5’-to-3’ direction, accounting for the length of input RNA fragments using MACS (2.1.1). UCSC tools 315 and RSeQC v. 2.6.1 were employed for bigwig format transformation and normalization to facilitate read coverage visualization and comparison across samples. Regions enriched in m^6^A were identified using the transcriptome based peak calling algorithm MeTPeak [25]. m^6^A peak summits were determined by defining the base position with the highest IP over input fragment ratio within the peak. For analyses of enriched motifs in summit regions, peak summits were extended in both directions to 200 nt and the top 2000 peaks were used for de novo motif discovery using DREME (MEME Suite v. 5.04). Peaks in mRNA were assigned to the following non-overlapping regions; TSS (downstream 200 nt), 5’UTR, CDS, stop-codon (centered 400 nt), and 3’UTR. Metagene plots for visualization of peak distribution along transcriptomic features were generated using deeptools v.2.3.4. Peak intersection analyses were performed with bedtools v.2.27.1.

Quantitative differential methylation analyses between sample groups were performed using RADAR [26]. IP and input sequencing reads were mapped to HG38 as paired ends with reference annotation GENCODE v.25 using STAR v.2.27.0. IP and input sequencing reads were divided into 100 nt bins and IP read counts were adjusted to overall gene expression level. Bins containing less than 15 reads were filtered out, and differential methylation was calculated with FDR = 0.1 andǀLog2FCǀ ≥ 0.5. GO term enrichment analyses were performed with G:profiler [27].

### Correlation analyses

Normalized IP read counts per bin adjusted for expression per individual (hereby referred to as methylation level) were extracted using the “extractIP” function in RADAR. Bin positions were converted to genomic locations based on the description of RADAR by using in-house Python scripts. Thereafter, bedtools v.2.27.1 was used to extract the relevant IP read counts from the lists of differentially methylated regions. Spearman correlation between bin methylation level and clinical variables (Table 1) was calculated with SPSS v.28. Spearman correlation with *p* < 0.05 was considered as nominally significant. All P-values are presented uncorrected for multiple testing and were considered to be of nominal statistical significance. Results were visualized as scatter plots for selected targets with GraphPad prism 9.4.1.

### RNA sequencing analyses

Input RNA from the meRIP-seq experiments were subjected to RNA-seq data processing. RNA seq-data processing was performed as previously described [28]. The pipeline (10.7490/f1000research.1119130.1) is publicly available in the provided GitLab repository (https://gitlab.com/epigen/rnaseqfastqtoreadcounts), version 0.1 was used for this analysis. Data normalisation and differential expression analysis were performed with DESeq2 in a paired manner; multi-factor design was used to account for subject and tissue type at the same time. Genes with ǀLog2FCǀ > 1 and *p* < 0.01 were considered differentially expressed. All analyses involving raw DNA sequences were performed in the Services for sensitive data (TSD) facilities, owned by the University of Oslo, operated and developed by the TSD service group at the University of Oslo, IT-Department (USIT).

## Results

### Transcriptome-wide m^6^A profiling in human adipose tissue reveals high overlap between subcutaneous and visceral depots

**Fig. 1:**
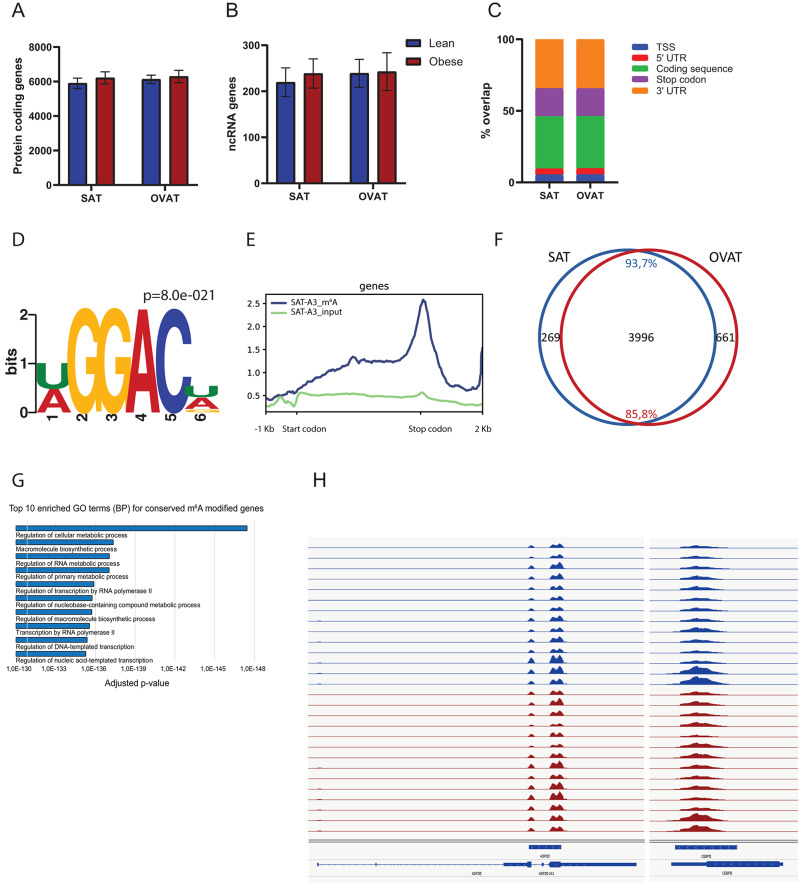
Global m^6^A profiles in human adipose tissue. Number of genes containing m^6^A peaks in SAT and OVAT from individuals with obesity (*n* = 10) and lean individuals (*n* = 3) in **A**: Protein coding genes and **B**: ncRNA. **C**: Genomic localization of m^6^A peaks in protein coding genes. TSS: TSS +200 nt, stop codon: stop codon ±200 nt. **D**: Enriched RRACH motifs in m^6^A peak summit regions exemplarily for one sample, SAT. **E**: Metagene profile plots for m^6^A peaks exemplarily for one sample, SAT. Plots show start codon, coding sequence, stop codon, 5’UTR (1 kb) and 3’UTR (up to 2 kb). Motifs and metagene profiles for all individuals and depots are shown in fig. S2. **F**: Venn diagram illustrating genes containing conserved peaks (intersecting in all 13 individuals) in SAT and OVAT and number of genes containing conserved peaks in both depots. **G**: Top 10 enriched GO terms (biological process) for conserved m^6^A modified genes, generated with gProfiler [27]. **H**: IGV browser shots of conserved m^6^A peaks in *ADIPOQ* and *CEBPD* gene regions. Figure shows strand specific, normalized enrichment of m^6^A across gene regions. AX: individuals with obesity; KX: lean individuals

To study the role of m^6^A in human adipose tissue, we performed global transcriptome-wide m^6^A profiling on intra-individually paired biopsies of abdominal subcutaneous (SAT) and omental visceral adipose tissue (OVAT) from 10 female individuals with morbid obesity (BMI ≥35 with comorbidities or BMI ≥40) and 3 female lean individuals (BMI < 25) using meRIP-seq. The main clinical characteristics of the study participants are shown in Table 1 (discovery cohort). A summary of mapping statistics for all samples is provided in Table S2. Using the RNA based peak caller MeTPeak [25], we identified on average ~12,000 m^6^A peaks in about 6,000 genes (*n* = 13, Fig. 1A, Table S3) in both types of adipose tissue depots. We also identified on average ~250 long non-coding RNAs (lincRNA) containing m^6^A (Fig. 1B) in SAT and OVAT (Table S3). Peak numbers were similar between individuals with obesity and lean subjects (Fig. 1A,B). We also did not observe enrichment of m^6^A in negative control regions, such as the *GAPDH* gene (Fig. S1). Validating the quality of the data, all samples showed enrichment of GGACH motifs or similar in peak summit regions and enrichment of m^6^A in stop codon regions as previously described [29, 30] (Fig. 1C–E; Table S4; Fig. S2). In addition to stop codon regions, m^6^A was also abundant in coding regions and in 3’UTR sequences (Fig. 1C). Collectively, when comparing peaks across samples, we find that m^6^A patterns are overall highly similar across depots and are also highly similar between lean subjects and individuals with obesity. We find that a high percentage (93,7%) of the peaks present in all SAT samples also are present in OVAT and vice versa (Fig. 1F). In total 5,976 m^6^A peaks across 3,996 genes were conserved across all individuals in both SAT and OVAT (Fig. 1F; Table S5). Genes containing such conserved peaks show enrichment of GO terms related to metabolic and transcriptional regulation (Fig. 1G), pointing towards an important role of m^6^A in gene regulation in adipose tissue in general. Examples of common adipose tissue m^6^A peaks are shown in *ADIPOQ*, CEBPA, *CEBPD and LEP* gene regions (Fig. 1H, Fig. S1), genes playing central roles in adipose tissue biology.

### Differential m^6^A methylation analysis identifies adipose tissue depot-specific targets

**Fig. 2:**
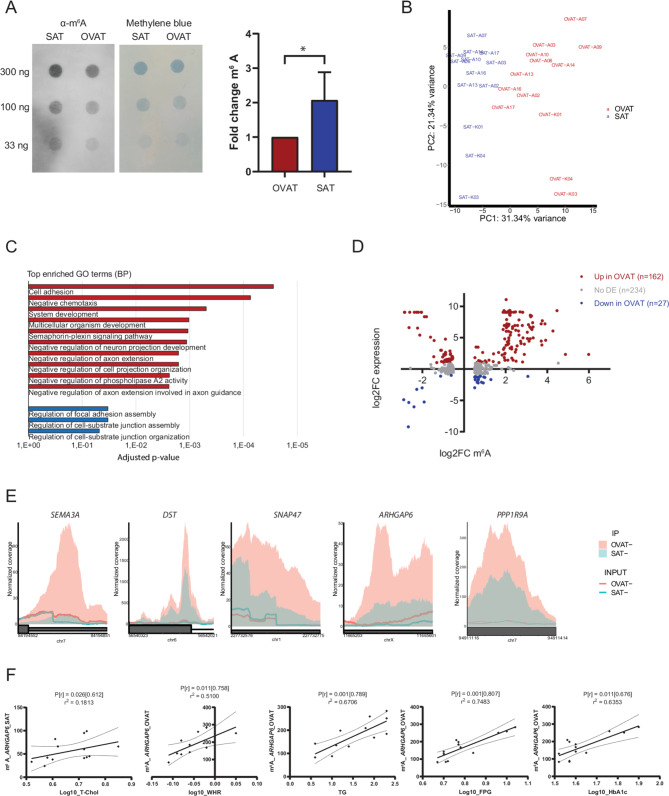
Depot specific m^6^A signatures between human SAT and OVAT. **A**: Representative image of m^6^A dot blots showing anti-m^6^A, methylene blue staining for SAT and OVAT and quantification of blots from 5 subjects. *: *p* < 0.05, paired t-test, all blots are shown in fig S3. **B**: Principle component analysis of global m^6^A levels, generated with RADAR. **C**: Most highly enriched GO terms (biological process (BP)) for SAT vs OVAT DMRs (table S7), genes hypermethylated in OVAT are shown in red (top 10 GOterms ranked by p-value), genes hypermethylated in SAT are in blue (adjusted p-value < 0.001), generated with gProfiler. **D**: Scatter plot of expression status (log2FCdetermined by RNA-seq) of genes with differentially methylated regions (DMRs, |log2FC| ≥ 1.5) in SAT vs. OVAT. **E**: Visual representation of selected DMRs shown in Table 2. Plots show mean normalized coverage in the associated region, *n* = 13. **F**: Significant correlations between normalized m^6^A read counts in DMR in *ARHGAP6* 5’UTR (chrX:11665203-1665601) region and clinical variables. P-value [Spearman’s rho] and r^2^ for the regression line is shown

We next sought to investigate whether m^6^A methylation confers depot-specific differences between SAT and OVAT. First, DotBlot analysis revealed significantly higher global m^6^A levels in SAT-derived mRNA compared to OVAT (Fig. 2A, Fig. S3), suggesting a depot-specific function. Further, principal component analysis (PCA) on global m^6^A levels in the meRIP-seq data (Fig. 2B), revealed a clear grouping based on the depot of origin. We next performed a quantitative analysis of the meRIP-seq data using RADAR [26] to identify differentially methylated targets. Indeed, we identified 430 differentially methylated regions (DMRs) (258/172 hypermethylated in OVAT/SAT; ǀLog2FCǀ ≥ 0.5, FDR < 0.1) in 339 genes between SAT and OVAT (Table 2, Table S6). To test for putative functional implications of the identified DMRs, we performed gene ontology (GO) analyses of the genes hypermethylated in the respective depots. Genes hypermethylated in OVAT in general showed an enrichment of GO-terms involved in cell adhesion, chemotaxis and development (Fig. 2C, Table S7). Of particular interest, we find enrichment of genes involved in the semaphorin-plexin signalling pathway with *SEMA3A, SEMA3B, SEMA3G, SEMA6D*, *PLXNA3* and *PLXNB1* all being hypermethylated in OVAT. Correspondingly, we also find semaphorin binding to be the most highly enriched GO term on molecular function (Table S7). Genes hypermethylated in SAT show lower numbers of enriched GO terms, such GO terms include genes involved in regulation of focal adhesions and cell-substrate binding (Fig. 2C, Table S7).

### Integrated analysis of differential m^6^A methylation with gene expression

We further investigated the relationship between differential m^6^A methylation and gene expression by integrating meRIP-seq with corresponding RNA-seq data (Table S8). Analysing the RNAseq data separately, we found a clear clustering based on the depot of origin (Fig S4A, D). We identified a total of 1476 differentially expressed transcripts between SAT and OVAT, of which 1025 were upregulated in OVAT and 452 were upregulated in SAT (Table S8). Differentially expressed genes were enriched in developmental, morphogenic, cell adhesion, and extracellular matrix pathways (Table S9), consistent with previous studies [31–34]. When integrating RNA-seq with meRIP-seq data, we found that around half of the DMRs (57%, *N* = 240) were located in genes that were not differentially expressed (Fig. 2D). A relatively large proportion of the genes showed upregulated gene expression in OVAT (37%, *N* = 157), whilst only a few were downregulated (6%, *N* = 26, Fig. 2D). Overall, no clear effect direction was observed between changes in m^6^A methylation and expression of the corresponding genes (chi-square test, *p* = 0.4553).

### m^6^A methylation levels in selected DMRs correlate with clinical variables of obesity

We further investigated whether m^6^A levels in the identified DMRs correlate with clinical variables related to obesity, fat distribution and metabolic parameters of glucose and insulin metabolism. Based on the differential methylation analysis, we focused on 5 specific DMRs. These targets were chosen from the top 50 identified DMRs (Table 2) based on expression status in adipose tissue (TPM ≥10 in SAT and OVAT) and were located in *ARHGAP6, DST*, *SNAP47*, *PPP1R9A* and *SEMA3A* gene regions, all showing elevated m^6^A in OVAT (Fig. 2E). We observed that m^6^A levels in the DMR in 5’UTR of *ARHGAP6*, encoding Rho GTPase activating protein 6, correlate with total cholesterol in SAT (Spearman correlation *p* < 0.05; Fig. 2G) and with waist to hip ratio (WHR), triglycerides (TG), fasting serum glucose (FSG) and HbA1c in OVAT (*p* < 0.05; Fig. 2H). Additionally, DMRs in *SEMA3A, SNAP47DST* and *PPP1R9A* gene regions show significant correlations with clinical variables (*p* < 0.05; Fig S5).

### Validation of depot specific m^6^A methylation for selected targets in a separate validation cohort

**Fig. 3:**
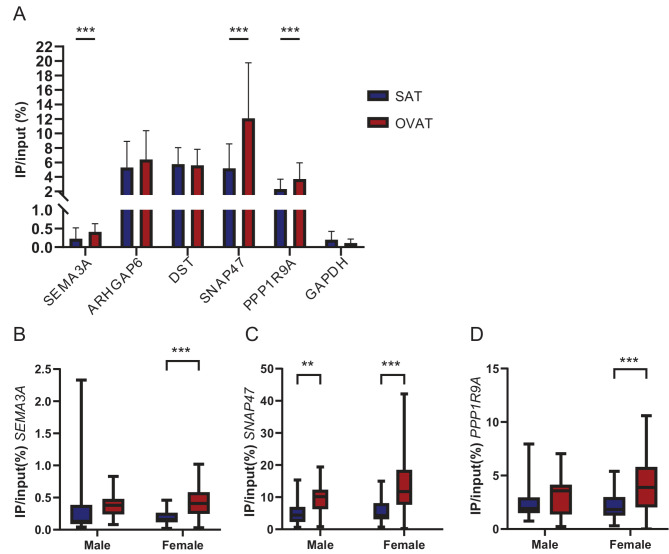
Depot specific m^6^A levels in validation cohort. **A**: meRIP-qPCR results from selected DMRs in *SEMA3A*, *ARHGAP6*, *DST*, *SNAP47*, *PPP1R9A* and *GAPDH* (negative control) results are shown as mean + SD, *n* = 72. B-D: meRIP-qPCR results grouped on gender for **B**: SEMA3A, **C**: SNAP47, **D**: PPP1R9A. Results are presented as boxplots with the horizontal line indicating the median and the box representing the interquartile range. Male: *n* = 25; female: *n* = 47; *: *p* < 0.05, **p < 0.01, ***p < 0.001, Wilcoxons signed rank test

To validate depot-specific m^6^A methylation found in the discovery cohort, we performed meRIP-qPCR on selected targets in a larger number of samples of paired samples of SAT and OVAT from individuals with obesity (validation cohort, *n* = 72, Table 1). We successfully validated depot-specific methylation for identified sites in *SNAP47, PPP1R9A* and *SEMA3A* (Fig. 3A). *ARHGAP6* shows similar trends as the validation cohort with elevated m^6^A in OVAT but does not reach the significance threshold (*p* = 0.052). We were not able to validate differential methylation of the *DST* coding region (Fig. 3A). In contrast to the discovery cohort, the validation cohort contained data from both male and female patients (Table 1). We therefore investigated the potential influence of gender on the observed differences in m^6^A. We observed that overall, the effect size of the depot specific differences is larger in the female compared to the male group (Fig. 3B–D). Further, only *SNAP47* was differentially methylated between SAT and OVAT in the male group (Fig. 3C), while the female group retained depot specific differences between *SNAP47, PPP1R9A* and *SEMA3A*. However, the same effect direction on m^6^A was observed in the male group, with trend of elevated m^6^A in OVAT for all three targets.

### Identification of obesity specific m^6^A methylation sites

**Fig. 4:**
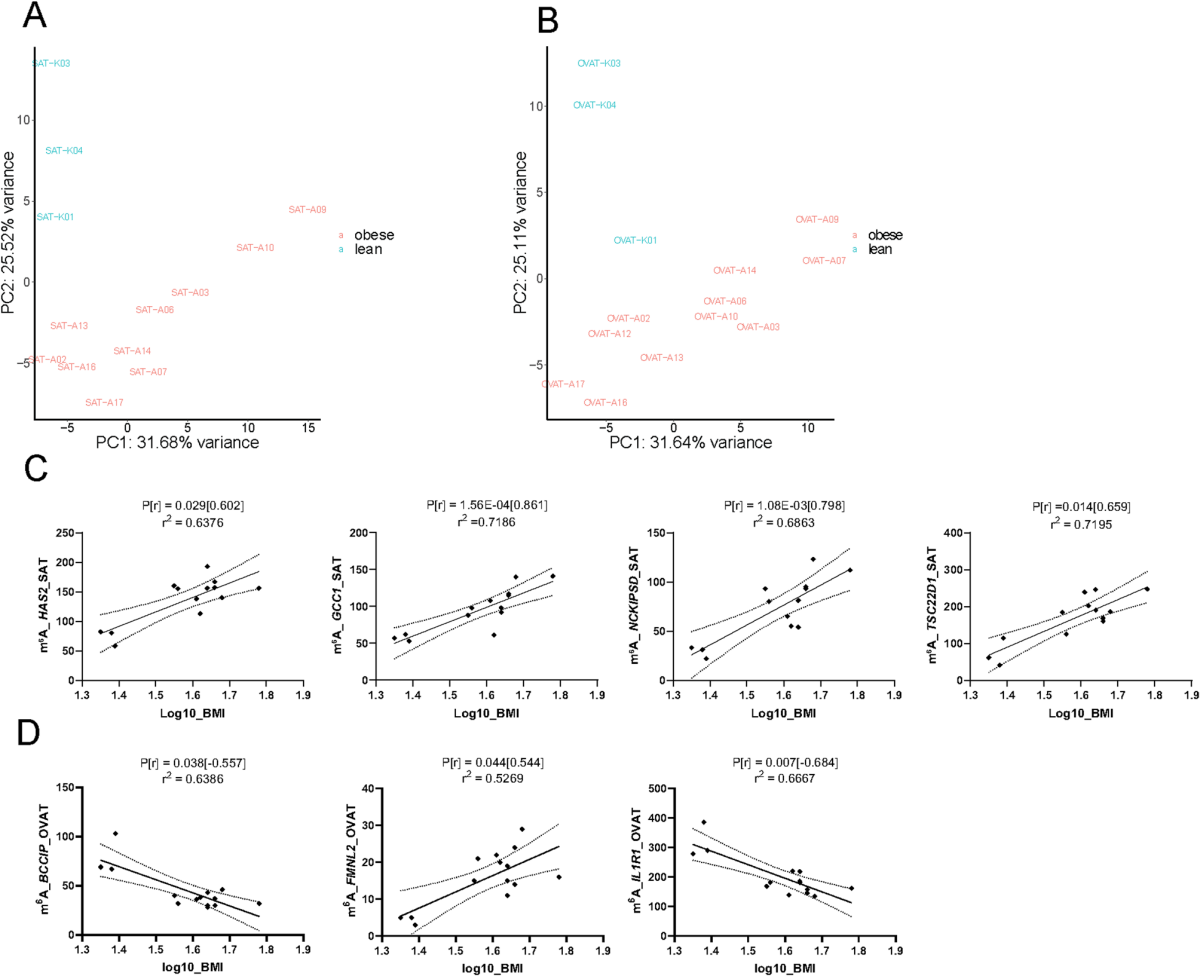
Obesity specific m^6^A signatures. **A, B**: Principle component analysis of global m^6^A levels in lean individuals (BMI ≤25) and subjects with obesity (BMI ≥35) in A: SAT and B: OVAT, generated with RADAR. **C, D**: Correlation of m^6^A level with BMI in selected DMRs in **C**: SAT: *GCC1*; chr7:127585149-127585248 (-), *HAS2*; chr8:121614211-121614310 (-), *TSC22D1;* chr13:44576257-44576356 (-), *NCKIPSD;* chr13:44576257-44576356 (-) and D: OVAT: *BCCIP*; chr10:125842277-125842376 (+), *FMNL2*; chr2:152618929-152619028(+), *IL1R1*; chr2:102176084-102176183(+). P-value [spearmans’ rho] and r^2^ for the regression line is shown

To further elucidate the role of m^6^A in obesity, we next investigated whether there are specific m^6^A DMRs in human adipose tissue that distinguish individuals with obesity (BMI ≥35 kg/m^2^) from their lean counterparts (BMI < 25 kg/m^2^). PCA revealed that samples clearly separated based on BMI status in both SAT and OVAT (Fig. 4A, B). For SAT, we identified 86 regions in 83 genes differentially methylated between lean and obese individuals (Table 3, Table S10). Correspondingly, in OVAT we observed 139 DMRs in 136 genes (Table 3; Table S10). Due to the relatively low number of genes, only a few significant GO terms were identified in our GO term analysis, all showing borderline significance for enrichment (Table S11). Further, we investigated in more detail selected DMRs in *TSC22D1, HAS2, GCC1, NCKIPSD* from SAT, all being hypermethylated in individuals with obesity and *BCCIP, IL1R1* (hypermethylated in lean) and *FMNL2* (hypermethylated in individuals with obesity) from OVAT (Table 3). We show that methylation of these targets correlates with BMI (Fig. 4C, D), further substantiating our results from the differential methylation analysis. For a number of these targets, we observed correlation with waist circumference as well as other clinical variables associated with fat distribution and obesity (Fig. S6A, B). We were not able to reproduce the observed correlation of *IL1R1* with BMI in the validation cohort (Fig. S6C). Further, we did not validate the other observed correlations of IL1R1 methylation with clinical variables in the validation cohort, however, similar to the discovery cohort, we observe an inverse relationship (although non-significant) between *IL1R1* m^6^A levels and waist circumference (Fig S6C). Additionally, when performing an integrated analysis of m^6^A methylation levels and gene expression in obesity specific DMRs, we observed that the majority of the obesity specific DMRs were not differentially expressed, neither in SAT nor in OVAT (Fig. S6D, E).

**Table 3:** Top 20 regions differentially methylated between patients with obesity and normal weight controls (*n* = 10/3) in SAT and OVAT, respectively, ranked by p-value. The complete list of differentially methylated regions is in table S10. Positive log2FC - hypermethylated in lean; negative Fold change – hypermethylated in obese

Gene name	Position (strand)	log2 Fold change	P-value	Genomic feature
*Subcutaneous adipose tissue*				
*CD2AP-DT*	chr6: 47,477,396-47477572 (-)	**1,8**	**3,83E-11**	ncRNA
*TSC22D1*	chr13: 44,576,257-44576356 (-)	**-1,69**	**5,50E-07**	5‘UTR
*SRPK2*	chr7: 105,142,346-105142445 (-)	**0,56**	**1,00E-06**	CDS
*CPED1*	chr7: 121,295,666-121295765 (+)	**0,58**	**1,06E-06**	3‘UTR
*HAS2*	chr8: 121,614,211-121614310 (-)	**-0,74**	**2,05E-06**	CDS
*UTP14C*	chr13: 52,030,836-52030935 (+)	**0,58**	**2,68E-06**	CDS
*GCC1*	chr7: 127,585,149-127585248 (-)	**-0,84**	**2,94E-06**	start codon
*SENP6*	chr6: 75,677,520-75677619 (+)	**0,55**	**4,39E-06**	intron
*NGFR*	chr17: 49,513,041-49513140 (+)	**-1,54**	**5,51E-06**	3‘UTR
*PAG1*	chr8: 80,976,747-80976846 (-)	**0,53**	**6,43E-06**	CDS
*TRDN*	chr6: 123,503,779-123503878 (-)	**1,06**	**7,36E-06**	CDS
*RP11-172H24.4*	chr13: 20,702,639-20702738 (-)	**1,17**	**7,95E-06**	ncRNA
*ADNP-AS1*	chr20: 50,944,392-50944491 (+)	**2,32**	**8,71E-06**	ncRNA
*NCKIPSD*	chr3: 48,674,432-48674531 (-)	**-1,06**	**9,35E-06**	3‘UTR
*RP11-172H24.4*	chr13: 20,703,129-20703228 (-)	**1,34**	**9,96E-06**	ncRNA
*S1PR2*	chr19: 10,223,823-10223922 (-)	**0,81**	**1,12E-05**	stop codon
*POSTN*	chr13: 37,563,278-37564525 (-)	**0,92**	**1,20E-05**	stop codon
*C8orf48*	chr8: 13,567,996-13568095 (+)	**-2,59**	**1,32E-05**	3‘UTR
*HOXD4*	chr2: 176,152,896-176152995 (+)	**0,76**	**1,62E-05**	3‘UTR
*CTD-2192J16.20*	chr19: 12,528,694-12577600 (-)	**-0,59**	**1,75E-05**	intron/CDS
*Omental visceral adipose tissue*				
*SMPD3*	chr16: 68,371,748-68371847 (-)	**0,77**	**7,49E-07**	CDS
*MIIP*	chr1: 12,022,104-12022203 (+)	**-2,57**	**9,70E-07**	CDS
*BCCIP*	chr10: 125,842,277-125842376 (+)	**0,8**	**1,45E-06**	3‘UTR
*ANKRD20A9P*	chr13: 18,835,667-18835766 (-)	**3,12**	**2,40E-06**	ncRNA
*PDE4DIP*	chr1: 148,982,649-148982748 (+)	**0,94**	**2,84E-06**	3‘UTR
*TIAF1*	chr17: 29,077,671-29077770 (-)	**-1,91**	**2,86E-06**	5‘UTR
*PTGER2*	chr14: 52,327,518-52327617 (+)	**0,78**	**3,27E-06**	3‘UTR
*TNKS1BP1*	chr11: 57,303,403-57308417 (-)	**0,91**	**3,67E-06**	intron
*NAA15*	chr4: 139,388,127-139388226 (+)	**1,62**	**4,63E-06**	3‘UTR
*ZFYVE9*	chr1: 52,239,033-52239132 (+)	**0,83**	**5,59E-06**	CDS
*SLC25A18*	chr22: 17,581,740-17581839 (+)	**2,94**	**5,97E-06**	intron
*FMNL2*	chr2: 152,618,929-152619028 (+)	**-1,46**	**6,22E-06**	CDS
*DHTKD1*	chr10: 12,120,934-12121033 (+)	**0,97**	**8,21E-06**	3‘UTR
*MIR3622A*	chr8: 27,703,191-27703290 (+)	**0,84**	**9,35E-06**	ncRNA
*IL1R1*	chr2: 102,176,084-102176183 (+)	**0,61**	**1,11E-05**	3‘UTR
*DCHS1*	chr11: 6,624,214-6624313 (-)	**-0,7**	**1,12E-05**	CDS
*TTC28*	chr22: 28,096,329-28098934 (-)	**1,07**	**1,15E-05**	CDS
*TBC1D30*	chr12: 64,875,566-64875665 (+)	**-1,9**	**1,40E-05**	CDS
*ZC3H12C*	chr11: 110,165,098-110165197 (+)	**1**	**1,45E-05**	CDS
*SOGA1*	chr20: 36,815,256-36815355 (-)	**-1,27**	**1,51E-05**	CDS

### A known FTO risk variant for obesity is not related to global m^6^A pattern

Because of the prominent and well described important role of genetic variants in the m^6^A eraser *FTO* in obesity [12], we also genotyped all individuals with obesity for the known *FTO* risk variant rs9939609 [11]. When comparing m^6^A levels between individuals homozygous for the risk variant (A/A genotype, *n* = 3) and homozygous carriers of the wild-type allele (T/T, *n* = 4), PCA analysis did not reveal any clustering of samples based on the *FTO* genotype (Fig. S7A, B). Moreover, we did not detect regions with differential methylation between the two groups (|log2FC| > 0.5, FDR < 0.1). Additionally, we did not observe differential expression of the *FTO* gene itself between carriers of the *FTO* risk allele and wild type carriers (Fig. S7 C, D). Taken together, we did not find clear association between *FTO* genotype and m^6^A methylation in adipose tissue. However, subtle differences at specific m^6^A sites may exist, that may become evident in larger population studies.

### Contribution of adipocytes to global m^6^A signatures in adipose tissue

**Fig. 5:**
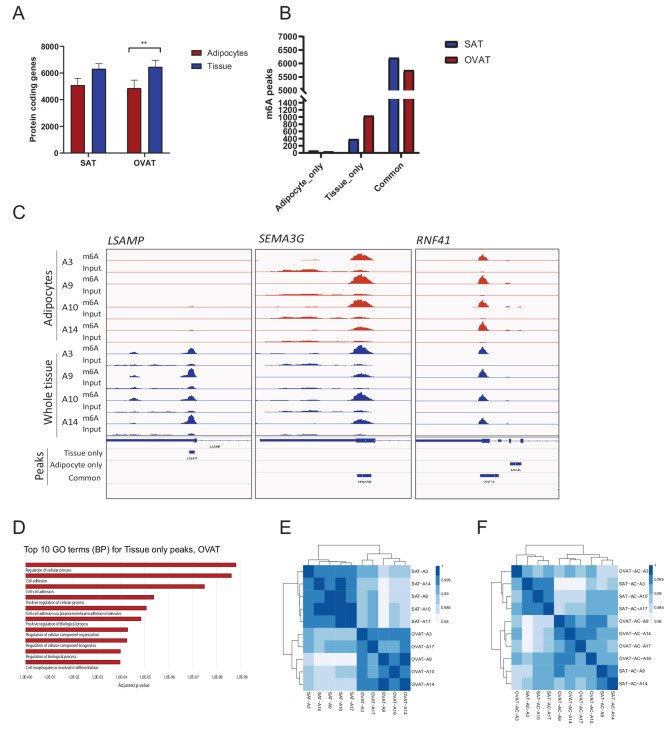
Contribution of adipocytes to overall m^6^A signatures in adipose tissue. **A**: Numbers of protein coding genes containing m^6^A in AC and in AT (paired samples, n=4, *: p<0.05 **: p<0.01, paired t-test). **B**: Peak numbers present exclusively in AT or AC (AC/AT only) or common in both groups. **C**: Example IGV profiles of m^6^A enrichment in selected regions from AT unique (*LSAMP*), AC-AT common (*SEMA3G*) and AC unique (*RNF41*) m^6^A peaks in OVAT. **D**: Top 10 enriched GO-terms (BP) in AT unique peaks in OVAT, generated with gProfiler. The full list of enriched GO-terms are in Table S13. **E, F**: Heatmap with hierarchical clustering of RNA-seq data in **E**: adipose tissue and **F**: primary adipocytes from corresponding individuals (paired samples, n=5), generated with DESeq2

Adipose tissue is a heterogeneous tissue, containing multiple cell types [35], which may contribute differentially to the overall m^6^A profiles. To address the influence of cell type composition on m^6^A profiles, we performed meRIP-seq on primary adipocytes from SAT and OVAT from a subset (*n* = 4) of the patients included in the discovery cohort. The adipocytes showed an overall lower number of genes containing m^6^A peaks compared to whole adipose tissue (Fig. 5A), both in subcutaneous and in visceral depots, although only statistically significant for OVAT. By performing a stringent peak intersection analysis, we identified a large number of peaks overlapping between all primary adipocytes and whole adipose tissue biopsy samples (Common peaks, *n* = 5750/6210 in SAT/OVAT, Fig. 5A, Table S12), suggesting an overall high contribution of adipocytes to the m^6^A landscape of adipose tissue. The genes containing common peaks show enrichment in GO terms involved in transcriptional and metabolic regulation, similar to what was observed in adipose tissue in general (Fig. 1f, Table S13, group 1,4). Further, a substantial number of peaks were found exclusively in tissue, not present in primary adipocytes (tissue only, Table S14, Fig. 5B), thereby likely contributed by non-adipocyte cells of the adipose tissue. The number of tissue unique peaks is almost 3-fold higher in OVAT compared to SAT (1,037 vs. 384 peaks, Fig. 5B), suggesting that non-adipocyte cells to a larger degree contribute to the m^6^A profile in OVAT compared to SAT. Interestingly,”tissue only” peaks in OVAT were highly enriched for GO-terms associated with cell adhesion (Fig. 5D), similar to what was observed for depot specific DMRs hypermethylated in OVAT (Fig. 2c, Table S7). Additionally, a low number of peaks (63 in SAT/41 in OVAT; Table S15) were present in adipocytes, but not in corresponding adipose tissue (AC-only). These peaks mainly represent lowly enriched peaks, as exemplified for the *RNF41* gene (Fig. 5C). Contrary to tissue-specific clustering observed in global transcriptomic profiles of whole adipose tissue, purified adipocytes show no depot-specific clustering, suggesting that non-adipocyte cells mediate depot-specific gene expression differences (Figs. 5E, 5F). Collectively, this data implies that for both m^6^A methylation and gene expression, variation in adipocytes themselves may be of less importance for depot specific variation compared to other cell types of the adipose tissue.

### m^6^A modification affects RNA stability of identified target genes

**Fig. 6:**
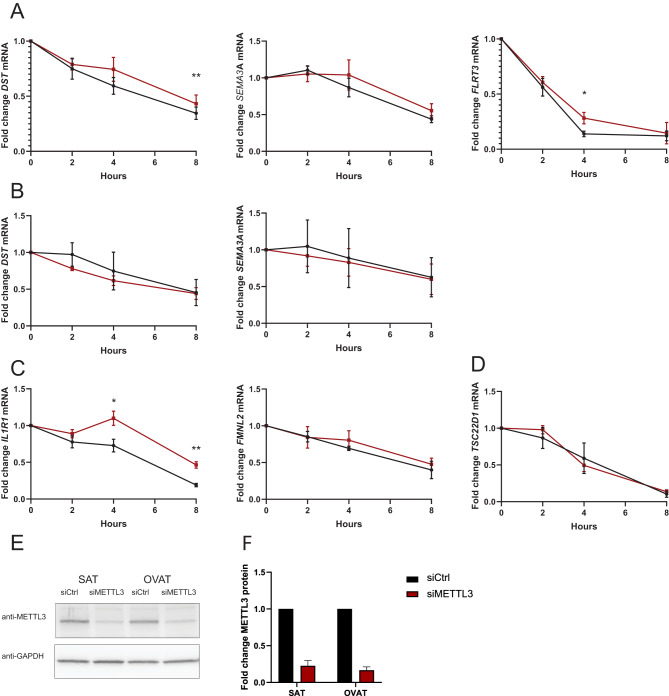
Influence of m6A on RNA stability of selected target genes identified in differential m6A analysis: **A-D**: RNA stability of EU labelled transcripts in cells transfected with siRNA targeting METTL3 (siMETTL3) or non-specific siRNA (siCtrl). **A-B**: Depot specific targets in **A**: OVAT **B**: SAT. **C-D**: Differentially methylated targets between lean and obese in **C**: OVAT and **D**: SAT. Results were normalized to *ACTB* and *GAPDH* expression. Results are presented as mean ± SEM, n=3 (*: p<0.05, ** :p<0.01; paired samples t-test). **E-F**: Validation of METTL3 Knockdown efficiency 72h after transfection by western blot. **E**: Representative immunoblot using anti-METTL3 and anti-GAPDH antibodies and **F**: quantification of METTL3 protein levels (n=3, mean ±SD)

The presence of m^6^A mRNA modifications may influence RNA metabolism in various ways, amongst others through regulation of RNA stability and decay. To study whether methylation of our identified targets influences RNA stability, we analysed decay of nascent RNA in cultured pre-adipocytes derived from SAT and OVAT in cells depleted of the m^6^A methyltransferase *METTL3*. Depot-specific targets investigated included *SEMA3A, DST* and *FLRT3* (all elevated m^6^A in OVAT, Fig. 6A, B). Interestingly, we show that in OVAT there is increased RNA stability for *DST* (8 h, *p* = 0.002) and *FLRT3* (4 h, *p* = 0.045)) in cells depleted of *METTL3* (Fig. 6A), suggesting that m^6^A promotes decay of these transcripts in OVAT. We also observe a trend of increased stability of *SEMA3A* upon *METTL3* depletion (Fig. 6A). In contrast, we observe no clear influence of *METTL3* depletion on RNA stability of *SEMA3A* and *DST* in SAT (Fig. 6B). Next, we investigated the impact of m^6^A on RNA stability of targets differentially methylated between individuals with obesity and lean subjects. These included *IL1R1* (hypermethylated among lean), *FMNL2* (hypermethylated in obesity) in OVAT (Table 3, Fig. 6C) and *TSC22D1* (hypermethylated in obesity) in SAT (Table 3, Fig. 6D). We show that *METTL3* knockdown clearly increases the stability of *IL1R1* in OVAT (4 h: *p* = 0.017; 8 h: *p* = 0.004), whilst there were no clear effects on stability of *FMNL2* (Fig. 5c). The obesity specific target *TSC22D1* in SAT (Fig. 6D) did not show altered RNA stability after *METTL3* depletion, suggesting that other mechanisms are involved in mediating functional effects of m^6^A for this target. In general, we show a trend of increased RNA stability of the selected targets upon METTL3 depletion in cells derived from OVAT, an effect which was not observed in cells from SAT.

In summary, we identify a catalogue of differentially methylated regions between SAT and OVAT and between lean individuals and subjects with obesity. Several of these targets show correlations between methylation levels and clinical variables related to obesity and fat distribution. We also find that OVAT contains a substantially higher number of m^6^A peaks originating from non-adipocyte cells as compared to SAT. Finally, we show that m^6^A deposition influences RNA lifetime of selected targets in human pre-adipocyte cell culture models.

## Discussion

Body fat distribution has an important role in metabolic health, and more knowledge on molecular properties of adipose tissue depots is warranted for developing better treatment options and prevention for obesity and its sequelae. Depot specific epigenetic marks and gene expression have been studied extensively [3, 4, 6, 36], however there are only limited data available elucidating epitranscriptomic regulation of gene expression in human adipose tissue [22]. Whether m^6^A methylation on RNA confers adipose tissue depot-specific effects, and whether and to what extent this correlates with clinical variables related to obesity is not yet established. We here present the first comparative transcriptome wide analysis of m^6^A in human adipose tissue - across different fat depots and between individuals with normal weight and with obesity, providing a valuable resource for further gene targeted and mechanistic studies. Our main findings are: **(i)** both global levels of m^6^A, and gene specific m^6^A signatures are adipose tissue depot-specific, **(ii)** m^6^A deposition at specific sites differs between lean individuals and individuals with obesity **(iii)** non-adipocyte cells contribute substantially to m^6^A signatures with almost three-fold more m^6^A peaks in OVAT and **(iv)** m^6^A has functional effects on selected target genes in cell culture models, impacting on RNA stability. **(v)** Finally, we show that m^6^A levels in selected targets correlate with clinical variables of obesity, fat distribution and glucose and insulin metabolism.

In line with previous studies [22], we show that the transcripts containing m^6^A are largely similar between different tissues and between individuals, but that there is a small catalogue of distinct m^6^A sites that differs between tissue types. We identified a list of adipose tissue depot specific DMRs that are particularly enriched in pathways associated with cell adhesion and developmental pathways. We also find enrichment of similar pathways in gene expression data, suggesting that m^6^A patterns to some extent reflect variability in gene expression. It is already well known that circulating levels of cell adhesion molecules are increased in obesity and that these may contribute to development of metabolic disease [37]. Cell adhesion molecules are also of importance in immune and inflammatory responses, consistent with the view of depot specific adipose tissue inflammation in OVAT contributing to metabolic disease [38]. We investigated in more detail several targets associated with cell adhesion, including *ARHGAP6* and *DST*. *ARHGAP6* encodes Rho GTPase-activating protein 6 and contains a DMR with elevated m^6^A in the 5’UTR region in OVAT. Methylation levels in this region in OVAT correlate with WHR, triglycerides, fasting serum glucose and HbA1c. *ARHGAP6* is to our knowledge not yet linked to a specific mechanism related to obesity or metabolic disease; however it was shown to be differentially DNA methylated in adipocytes in obese mice [39], and was reported to be involved in regulation of glucose metabolism, cell proliferation and migration by regulating STAT3 signalling in cancer [40]. *DST* encoding dystonin, a member of the plakin protein family, and a prominent cytoskeletal protein was hypermethylated in OVAT in our study. We show in functional analyses in preadipocytes that m^6^A modification on *DST* in OVAT leads to increased mRNA decay that may confer important functional implications. Indeed, we find correlation of *DST* m^6^A levels with several clinical variables including waist, triglyceride levels and fasting insulin. These results are in concordance with previous research showing that dystonin is differentially expressed between individuals with obesity and lean counterparts [41]. Dystonin has also been implicated in cardiomyopathy [42]. Interestingly, dystonin was observed to be an important player in the autophagosome-endolysosome pathway [43], which may represent a link to obesity. In line with this, genetic variants in *DST* were associated with body height, an anthropometric measure important for obesity [44].

Of additional interest, for DMRs hypermethylated in OVAT, we find enrichment of genes associated with semaphorin-plexin signalling pathways in GO-term analyses, including *SEMA3A, SEMA3B, SEMA3G, SEMA6D, PLXNA3* and *PLXNB1.* Recent evidence points towards a role of semaphorin-plexin signalling in adipogenesis, adipose tissue inflammation and metabolism [45]. Interestingly, semaphorins are also important players in monogenic obesity being involved in neuronal projection of pro-opiomelanocortin (POMC) neurons and thereby, contributing to neuronal circuitry in the leptin-melanocortin pathway [46, 47]. In our analysis, we identified a differentially methylated region in the *SEMA3A* 5’UTR region. We find that m^6^A levels at *SEMA3A* in OVAT correlates with triglycerides, waist and WHR. In line with this, genetic variation in the *SEMA3A* gene was shown to be associated with waist circumference in GWAS studies [48] and body height [49] adding further weight to the role of *SEMA3A* in adipose tissue.

Strengthening the results from the discovery cohort, we validate depot specific m^6^A levels at specific DMRs for 3 of 5 examined targets in a larger cohort of individuals with obesity (*n* = 72). These include *SEMA3A*, *Synaptosome associated protein 47 (SNAP47)* and *Protein Phosphatase 1 Regulatory Subunit 9A (PPP1R9A)*, all hypermethylated in OVAT. *SNAP47* and *PPP1R9A* are associated with neuronal pathways and have to our knowledge not yet been studied in the context of obesity. *SNAP47* was originally identified as a part of neuronal SNARE proteins associated with membrane fusion [50]. It is ubiquitously expressed, and recent evidence also support a role for *SNAP47* in regulation of autophagic flux [51, 52], which represent a potential link to obesity. *PPP1R9A* encodes the protein neurabin 1, which is a key regulator of protein phosphatase 1 activity and reorganization of the actin cytoskeleton and highly expressed in synapses [53]. Polymorphisms in *PPP1R9A* and the linked *PEG10* locus were associated with fat deposition in pigs [54], hinting at a potential role in human adipose tissue biology.

Intriguingly, when looking at depot specific differences in global m^6^A levels, by m^6^A dot blot, we find that SAT contains a higher level of m^6^A in mRNA compared to OVAT. This is somewhat contradictory to the results from the target specific differential methylation analysis discussed above, where most of the targets are hypermethylated in OVAT. However, the techniques used cannot be compared directly. The 339 genes containing DMRs identified in the sequencing-based analysis only represent a small fraction of the overall gene pool and do not necessarily make a large contribution to the global m^6^A level. The observation of increased number of hypermethylated targets in OVAT is also consistent with our corresponding RNA-seq data, which show an elevated number of genes with upregulated expression in OVAT compared to SAT, consistent with previously published data [4, 55]. Interestingly, global differences as well as target specific differences in m^6^A may reflect differences in cell composition in the respective depots [56]. Recent studies have revealed the presence of distinct populations of immune cells, adipocytes and progenitor cells in visceral and subcutaneous depots [35–58] that likely influence progression of obesity related comorbidities. Indeed, a recent paper deciphering cell type composition from bulk RNA-seq data comparing SAT and OVAT from the GTEX database, find that many of the genes upregulated in OVAT compared to SAT are not expressed in adipocytes, rather in adipose progenitor or cells of mesothelial origin [55]. This is consistent with our finding that depot specific expression patterns are likely contributed by other cells than primary adipocytes. Indeed, we see similar trends in the m^6^A data, showing a three-fold larger number of non-adipocyte peaks in OVAT compared to SAT.

In addition to the depot specific targets, we also observed differentially methylated transcripts between individuals with and without obesity. Examples of targets with differential methylation in SAT between individuals with obesity and lean counterparts include *Transforming Growth Factor β 1-stimulated Clone 22 D1* (*TSC22D1*), *Hyaluronan Synthase 2* (*HAS2*), *GRIP And Coiled-Coil Domain Containing 1* (*GCC1*) and *NCK Interacting Protein With SH3 Domain* (*NCKIPSD*). Several of these targets have previously been linked with obesity or metabolism. For instance, *TSC22D1* was shown to be involved in regulating cholesterol homeostasis in liver [59]. *Hyaluronan synthase 2* (*HAS2*) was shown to have an inhibitory role in adipogenic differentiation, influencing *PPARG* expression. Additionally, circulating hyaluronan was shown to negatively correlate with BMI and triglyceride levels [60]. The obesity specific DMRs from OVAT investigated in more detail include *Interleukin 1 Receptor 1* (*IL1R1*), *BRCA2 and CDKN1A Interacting Protein* (*BCCIP*) and *Formin Like 2* (*FMNL2).* Interestingly, we show that obesity specific methylation of *IL1R1* in OVAT correlates with clinical features of glucose metabolism such as serum triglycerides and HbA1c, and that m^6^A at this target promotes RNA decay in OVAT. This is in concordance with previous findings showing important roles of *IL1R1* in glucose homeostasis and adipogenesis in mouse models in response to high fat diet [61]. In support of this, it was shown that *IL1R1* expression was downregulated in OVAT of individuals with obesity and type 2 diabetes compared to non-diabetic controls [62]. The results presented here may suggest a novel regulatory mechanism of *IL1R1* expression, based on the regulatory effect on RNA stability imposed by m^6^A. *BCCIP* has been implicated in DNA double strand break repair [63], and it was shown to be required for nuclear localization of p21, which is a major regulator of senescence [64]. Adipose tissue senescence is implicated in disease development [65], and *BCCIP* might be involved in this process.

When correlating m^6^A with gene expression data, we find that the majority of the differentially methylated targets are in genes that are not differentially expressed, in particular for obesity specific DMRs. Additionally, we observe that the differentially methylated genes between SAT and OVAT to some extent mirror differentially expressed genes with an elevated number of upregulated genes in OVAT compared to SAT in both expression and methylation data. So far, for the targets with upregulated expression, it is unclear whether increased mRNA expression per se leads to an increased deposition of m^6^A or whether m^6^A contributes to higher mRNA levels. Our finding that many differentially methylated genes are not differentially expressed, suggests that m^6^A modification of these genes may influence aspects of RNA metabolism not detectable at transcriptional level, leading to differential protein levels or function. M^6^A may influence a plethora of different mechanisms, such as pre-mRNA processing, mRNA nuclear export, mRNA stability, translational initiation and efficiency, which would not necessarily impact overall mRNA levels [66]. These genes provide novel targets, which would not be discovered using gene expression data alone. In this work, we provided functional evidence that m^6^A impacts the rate of RNA decay of selected targets including *DST*, *FLRT3* and *IL1R1* in pre-adipocytes. The rate of decay is reduced in all three targets after METTL3 depletion in cells derived from OVAT. This suggests that m^6^A accelerates RNA turnover when present on these targets. We did not observe effects on RNA stability in cells derived from SAT, suggesting that functional effects of m^6^A may be depot-specific. In addition, the biological consequence of m^6^A is highly dependent on the localization of the modification and the associated reader proteins [10]. With regards to reader proteins, we previously showed that the m^6^A reader *YTHDC1* is differentially expressed between SAT and OVAT [17]. This reader is involved in several stages of RNA processing such as splicing, polyadenylation and nuclear export [66] and could contribute to adipose tissue depot-specific effects. Further studies are warranted to investigate, which specific reader proteins are involved in mediating such effects, and whether results translate to protein levels.

Although being the first study of its kind on a relatively large number of individuals, our study has some limitations. First, the method and antibody used to identify m^6^A is unable to distinguish between m^6^A and 2’-O-dimethyladenosine (m^6^A_m_), a RNA modification known to be involved in mRNA 5’ cap modification [67]. The results obtained here are likely a combination of both RNA modifications. This is important to consider for further functional studies of target genes identified in this study, especially for targets present in the 5’UTR region. m^6^A_m_ targets identified could also be relevant markers of obesity, as a role for m^6^A_m_ in metabolic dysregulation in obesity was recently shown [68]. In addition, both m^6^A and m^6^A_m_ are targets for FTO demethylation, making both modifications relevant in the obesity context [67]. Second, our discovery cohort includes only women. Due to known gender-specific differences in adipose tissue distribution and fat metabolism [69], and a limited sample size, we chose to focus on female individuals only, to minimize variability in the datasets and thereby strengthen the power of the results. This needs to be taken into consideration when interpreting the results. However, results from the larger validation cohort including both male and female patients with obesity, suggest that identified depot-specific differences may also be found in male patients. Finally, for the obesity specific analysis, the low number of lean individuals included could influence the results. The results from obesity specific analyses should therefore be interpreted with caution compared to the results from the depot specific analyses, which are based on a higher number of individuals, as well as a paired sample design.

## Conclusions

Taken together, our study provides novel targets implicated in obesity along with potentially novel regulatory mechanisms imposed by m^6^A. We identify a catalogue of differentially methylated targets between SAT and OVAT, as well as genes differentially methylated between lean individuals and patients with obesity. These targets may potentially serve as predictive biomarkers for metabolic disease. The data represent an important resource for future studies on the role of m^6^A in human adipose tissue and its relevance for clinical features of obesity.

## Electronic supplementary material

Below is the link to the electronic supplementary material.

Supplementary Material 1

Supplementary Material 2

Supplementary Material 3

## Data Availability

All the non-sensitive data supporting the final results and major findings of the study is included in the form of figures and tables in the main article as well as in the supplementary files. The NGS datasets generated and analysed during the current study are not publicly available due to data sensitivity. Please contact the corresponding author to discuss the possibilities of the controlled access to the datasets.

## Abbreviations

m^6^A: N^6^-methyladenosine
SAT: Subcutaneous adipose tissue
OVAT: Omental visceral adipose tissue
AC: Adipocyte
SVF: Stromal vascular fraction
DMR: Differentially methylated region
PCA: Principal component analysis
lincRNA: Long non-coding RNA
