# Supplementary material for: Global mapping of RNA N6-methyladenosine (m6A) in human subcutaneous and visceral adipose tissue reveals novel targets that correlate with clinical variables of obesity
Source: Biomark Res. 2025 Nov 12;13:146. doi: 10.1186/s40364-025-00857-0 (PMC12613671; doi:10.1186/s40364-025-00857-0)
Supplement: Supplementary file 2 — Supplementary Material 2 [file 40364_2025_857_MOESM2_ESM.pdf]

Supplementary table legends

Table S1: Primer sequences used in this study.

Table S2: Mapping statistics for meRIPseq data analysis.

Table S3: Peak calling summary and statistics.

Table S4: Enriched Motifs in m^6A peak summits.

Table S5: List of conserved m^6A peaks: Peaks present in all replicates, SAT and OVAT.

Table S6: List of differentially methylated regions (DMRs; |logFC| > 0.5, fdr <0.1) between SAT and OVAT and associated gene expression changes.

Table S7: Enriched GO terms for SAT vs. OVAT DMRs.

Table S8: List of differentially expressed genes between SAT and OVAT, |logFC| > 1, padj < 0.01.

Table S9: Enriched GO terms – differentially expressed genes, SAT vs. OVAT

Table S10: List of identified DMRs (|logFC| > 0.5, fdr <0.1) between lean and obese individuals and associated gene expression changes.

Table S11: Enriched GO terms - lean vs. obese DMRs

Table S12: List of common m^6A peaks between adipocytes and adipose tissue.

Table S13: Enriched GO terms in gene lists from adipocyte vs. adipose tissue intersection analysis.

Table S14: List of adipose tissue unique m^6A peaks (not present in adipocytes).

Table S15: List of adipocyte unique m^6A peaks (not present in adipose tissue).

Supplementary figure legends

Figure S1: Visualization of selected regions positive and negative for m^6A in human adipose tissue. Strand specific IGV profiles of m^6A IP and corresponding input samples in adipogenic genes from SAT and OVAT of 3 individuals with obesity. «SAT-VAT conserved» denote peaks overlapping between all adipose tissue samples (n=26 (13 individuals, SAT, OVAT)). *GAPDH* is included as a negative control, showing no m^6A enrichment.

Figure S2: Quality control of m^6A peak data: Enrichment of RRACH motifs and metagene profiles in peak summit regions for: A: adipose tissue (AT), B: adipocytes (AC). Motif analysis was performed with MEME, adjusted p-value is shown.

Figure S3: m^6A dot blot results: 5 intra-individually paired samples of SAT and OVAT from individuals with obesity. Results show anti-m^6A immunoblots and methylene blue as loading control.

Figure S4: Differential gene expression analysis by RNAseq. A-C: PCA analysis and D-F: hierarchical clustering for A, D: SAT vs. OVAT (n=13), B, E: Lean vs. obese in SAT (n=3/10), C, F: Lean vs. obese in OVAT (n=3/11). Differential expression was defined as $|Log2FC| > 1$ and adjusted p-value < 0.01.

Figure S5: Significant correlations of depot specific DMRs (SAT vs. OVAT) with clinical parameters. m^6A levels in DMRs of **A:** *SEMA3A*: chr7;84194552-84194851 (-), **B:** *SNAP47*; chr1:227732576-227732775 (+), **C:** *DST*; chr6:56540823-56541121 (-) **D:** *PPP1R9A;* chr7:94911116-94911414 (+). P-value [Spearmans' rho] and r^2 for the regression line is shown.

Figure S6: Correlations and meRIP-qPCR for obesity specific DMRs (normal weight vs. obesity). Significant correlation of m^6A level with clinical traits in discovery cohort in selected DMRs in **A:** SAT: *GCC1*; chr7: 127585149-127585248(-), *HAS2*; chr8: 121614211-121614310(-), *TSC22D1;* chr13: 44576257-44576356(-), *NCKIPSD;* chr13: 44576257-44576356(-) and **B:** OVAT: *IL1R1*; chr2: 102176084-102176183(+).**C:** Correlations of m^6A levels measured in *IL1R1* DMR region with BMI and waist circumference in validation cohort (meRIP-qPCR, n=72). P-value [Spearmans' rho] and r^2 for the regression line is shown. **D, E:** Scatter plot of expression status (log2FC determined by RNAseq) of genes with differentially methylated regions ($|log2FC| \geq 1.5$, FDR < 0.1) between lean and obese in **E:** SAT and **F:** OVAT.

Figure S7: Differential m^6A and gene expression analysis in *FTO* risk allele rs9939609 carriers. A, B: PCA plot of global methylation level in **A:** SAT and **B:** OVAT, generated by RADAR, subjects grouped by genotype (individuals with obesity homozygous for risk or wild type variant). **C, D:** Expression of *FTO* (RNA-seq data, transcript per million (TPM)) grouped on rs9939609 genotype in **C:** SAT, **D:** OVAT. rs9939609 risk allele: A, wild-type allele: T.

Figure S1

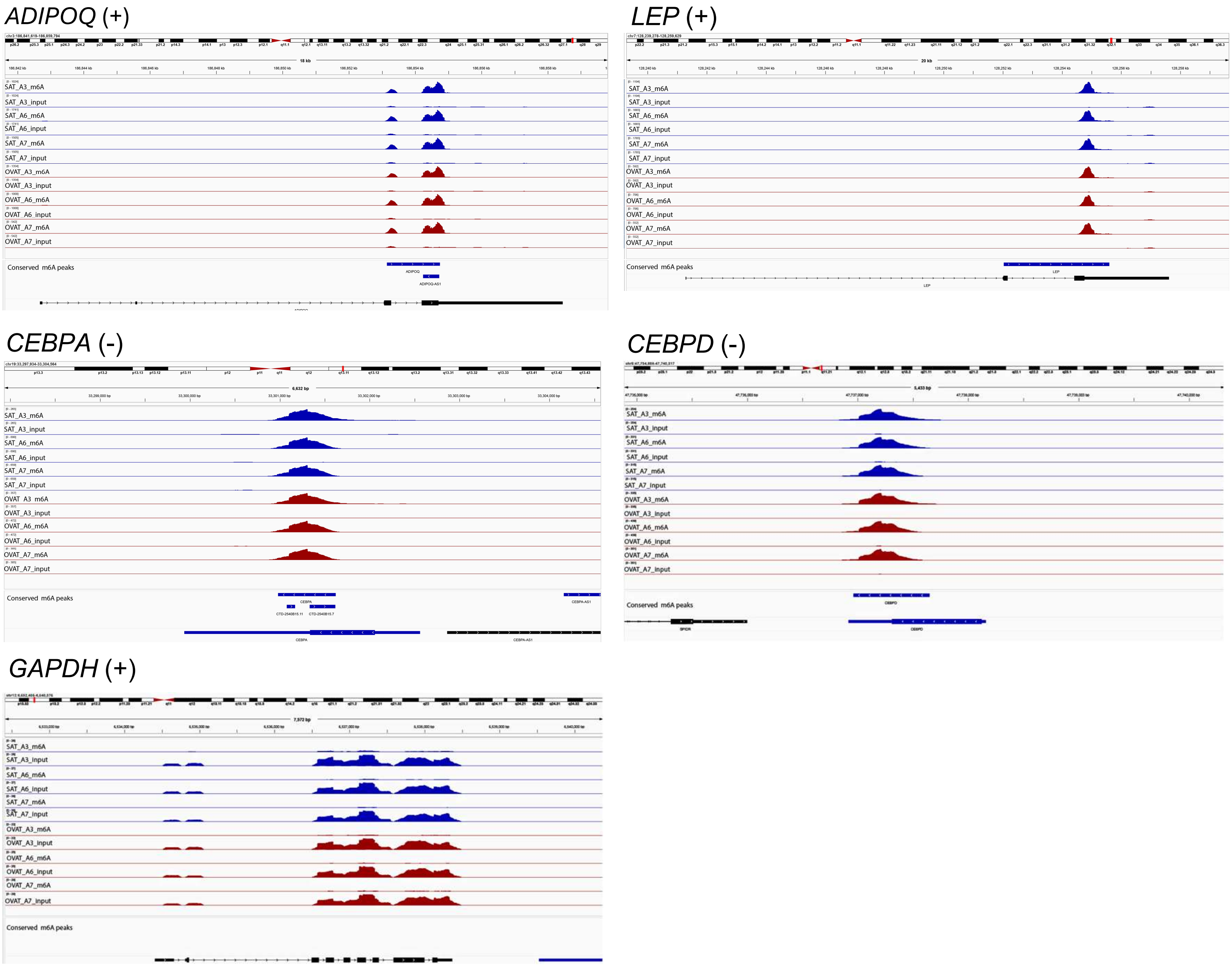

ADIPOQ (+)
SAT_A3_m6A
SAT_A3_input
SAT_A6_m6A
SAT_A6_input
SAT_A7_m6A
SAT_A7_input
OVAT_A3_m6A
OVAT_A3_input
OVAT_A6_m6A
OVAT_A6_input
OVAT_A7_m6A
OVAT_A7_input
Conserved m6A peaks
LEP (+)
SAT_A3_m6A
SAT_A3_input
SAT_A6_m6A
SAT_A6_input
SAT_A7_m6A
SAT_A7_input
OVAT_A3_m6A
OVAT_A3_input
OVAT_A6_m6A
OVAT_A6_input
OVAT_A7_m6A
OVAT_A7_input
Conserved m6A peaks
CEBPA (-)
SAT_A3_m6A
SAT_A3_input
SAT_A6_m6A
SAT_A6_input
SAT_A7_m6A
SAT_A7_input
OVAT_A3_m6A
OVAT_A3_input
OVAT_A6_m6A
OVAT_A6_input
OVAT_A7_m6A
OVAT_A7_input
Conserved m6A peaks
CEBPD (-)
SAT_A3_m6A
SAT_A3_input
SAT_A6_m6A
SAT_A6_input
SAT_A7_m6A
SAT_A7_input
OVAT_A3_m6A
OVAT_A3_input
OVAT_A6_m6A
OVAT_A6_input
OVAT_A7_m6A
OVAT_A7_input
Conserved m6A peaks
GAPDH (+)
SAT_A3_m6A
SAT_A3_input
SAT_A6_m6A
SAT_A6_input
SAT_A7_m6A
SAT_A7_input
OVAT_A3_m6A
OVAT_A3_input
OVAT_A6_m6A
OVAT_A6_input
OVAT_A7_m6A
OVAT_A7_input
Conserved m6A peaks

Figure S2A

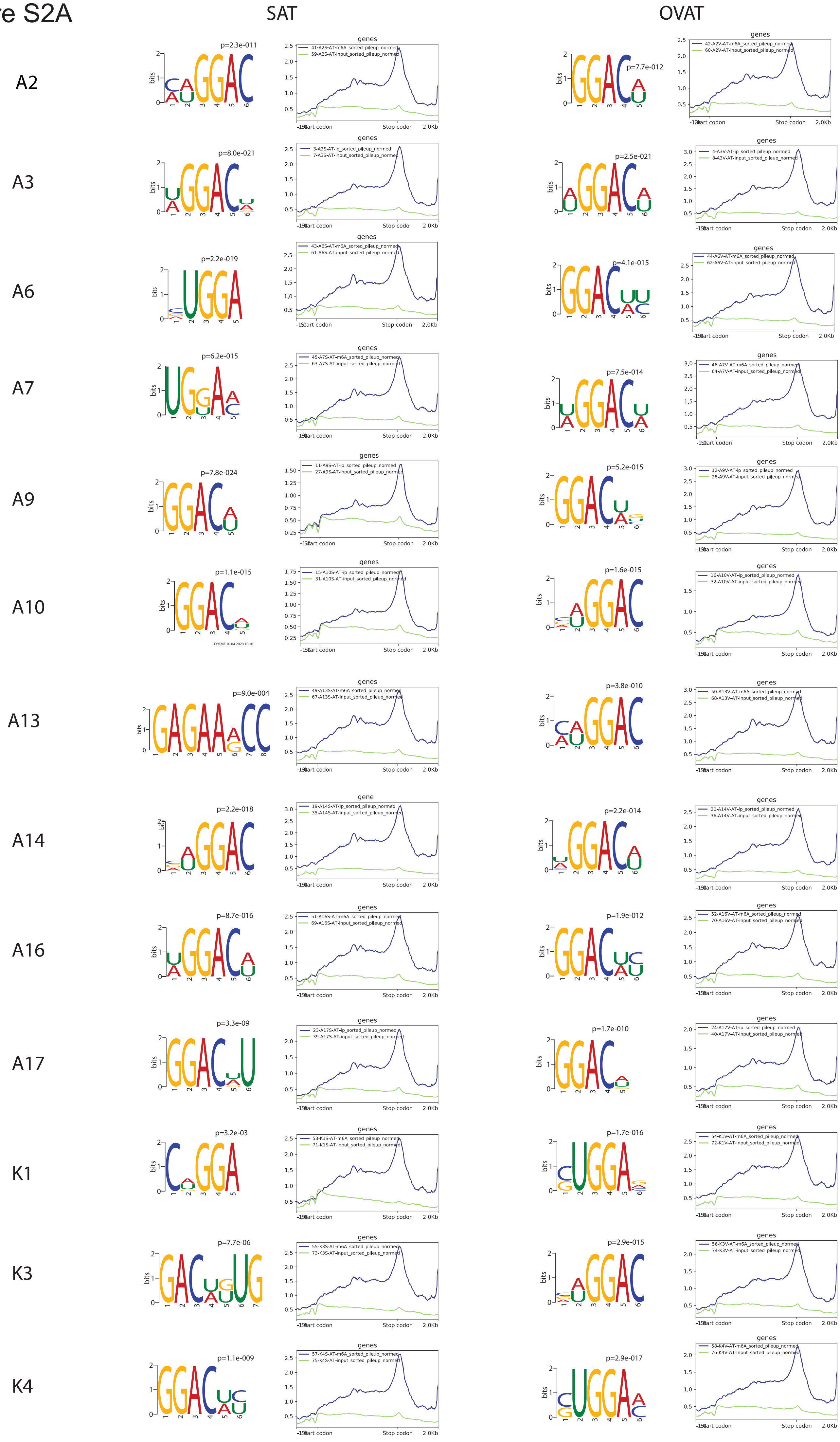
SAT
OVAT
A2
p=2.3e-011
p=7.7e-012
A3
p=8.0e-021
p=2.5e-021
A6
p=2.2e-019
p=4.1e-015
A7
p=6.2e-015
p=7.5e-014
A9
p=7.8e-024
p=5.2e-015
A10
p=1.1e-015
p=1.6e-015
A13
p=9.0e-004
p=3.8e-010
A14
p=2.2e-018
p=2.2e-014
A16
p=8.7e-016
p=1.9e-012
A17
p=3.3e-09
p=1.7e-010
K1
p=3.2e-03
p=1.7e-016
K3
p=7.7e-06
p=2.9e-015
K4
p=1.1e-009
p=2.9e-017
genes
Stop codon
2.0Kb
bits

Figure S2B

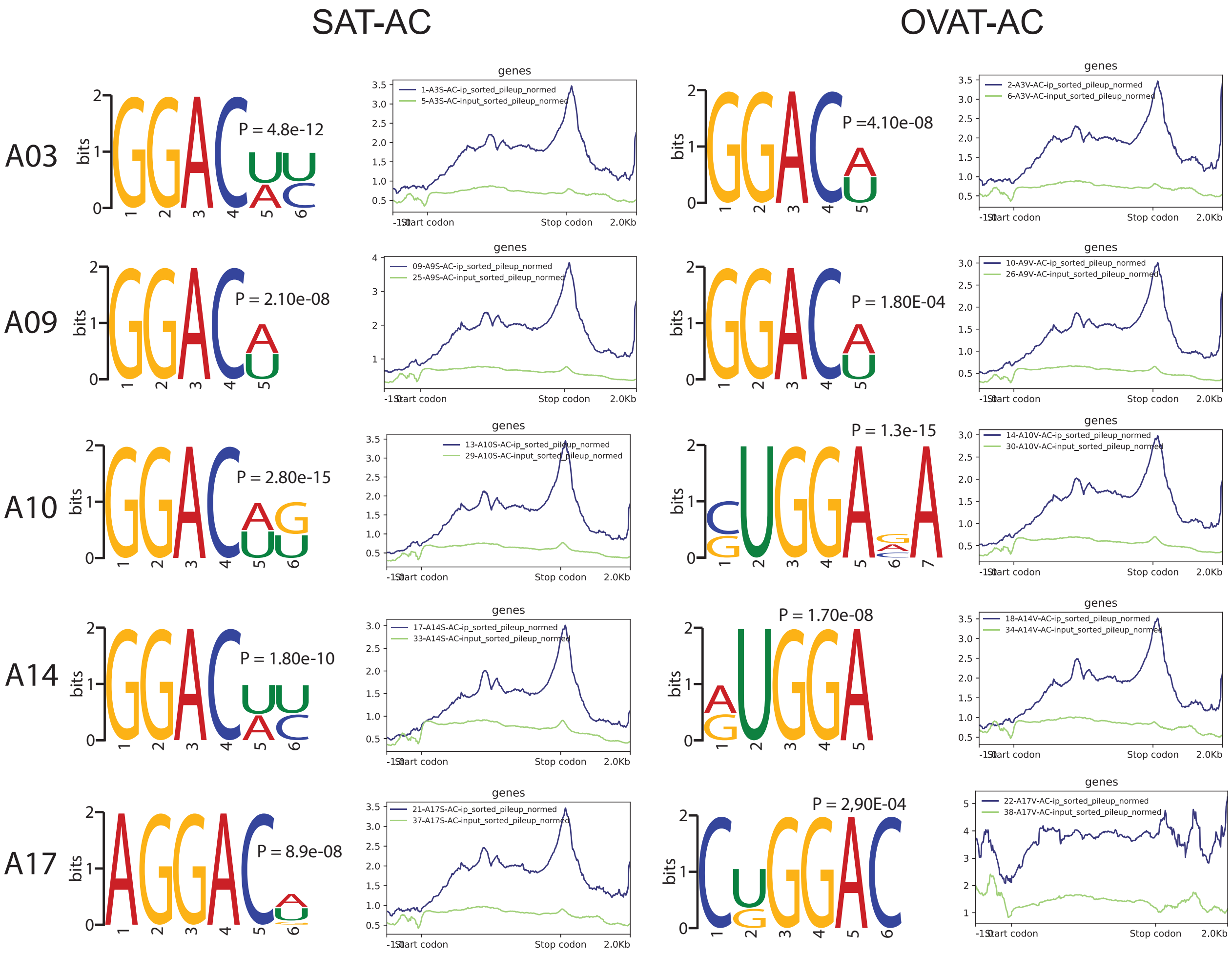
SAT-AC
OVAT-AC
A03
A09
A10
A14
A17
bits
P = 4.8e-12
P =4.10e-08
P = 2.10e-08
P = 1.80E-04
P = 2.80e-15
P = 1.3e-15
P = 1.80e-10
P = 1.70e-08
P = 8.9e-08
P = 2,90E-04
genes
Start codon
Stop codon
2.0Kb

Figure S3

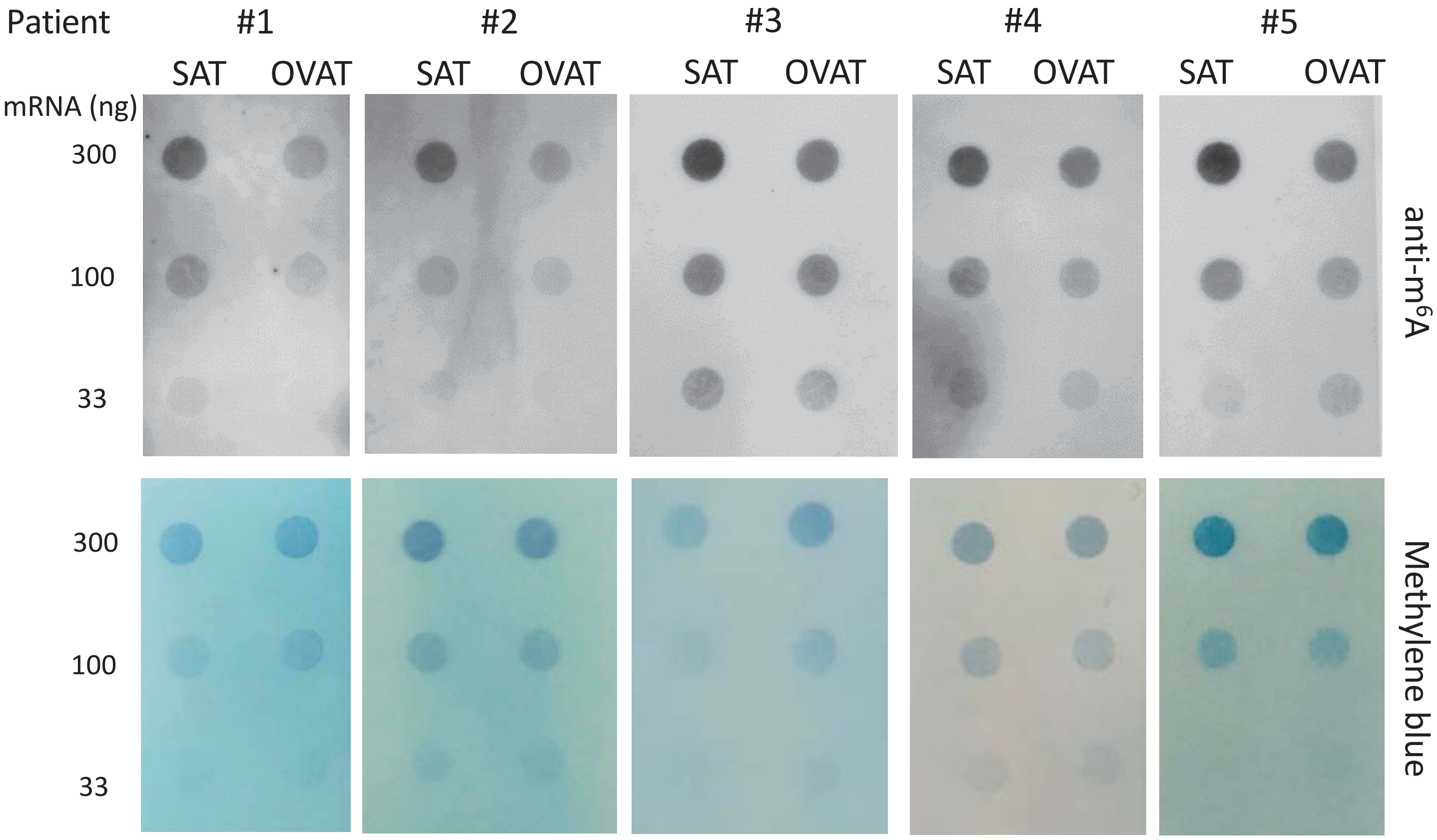
Patient
#1
#2
#3
#4
#5
SAT
OVAT
SAT
OVAT
SAT
OVAT
SAT
OVAT
SAT
OVAT
mRNA (ng)
300
100
33
anti-m6A
300
100
33
Methylene blue

Figure S4

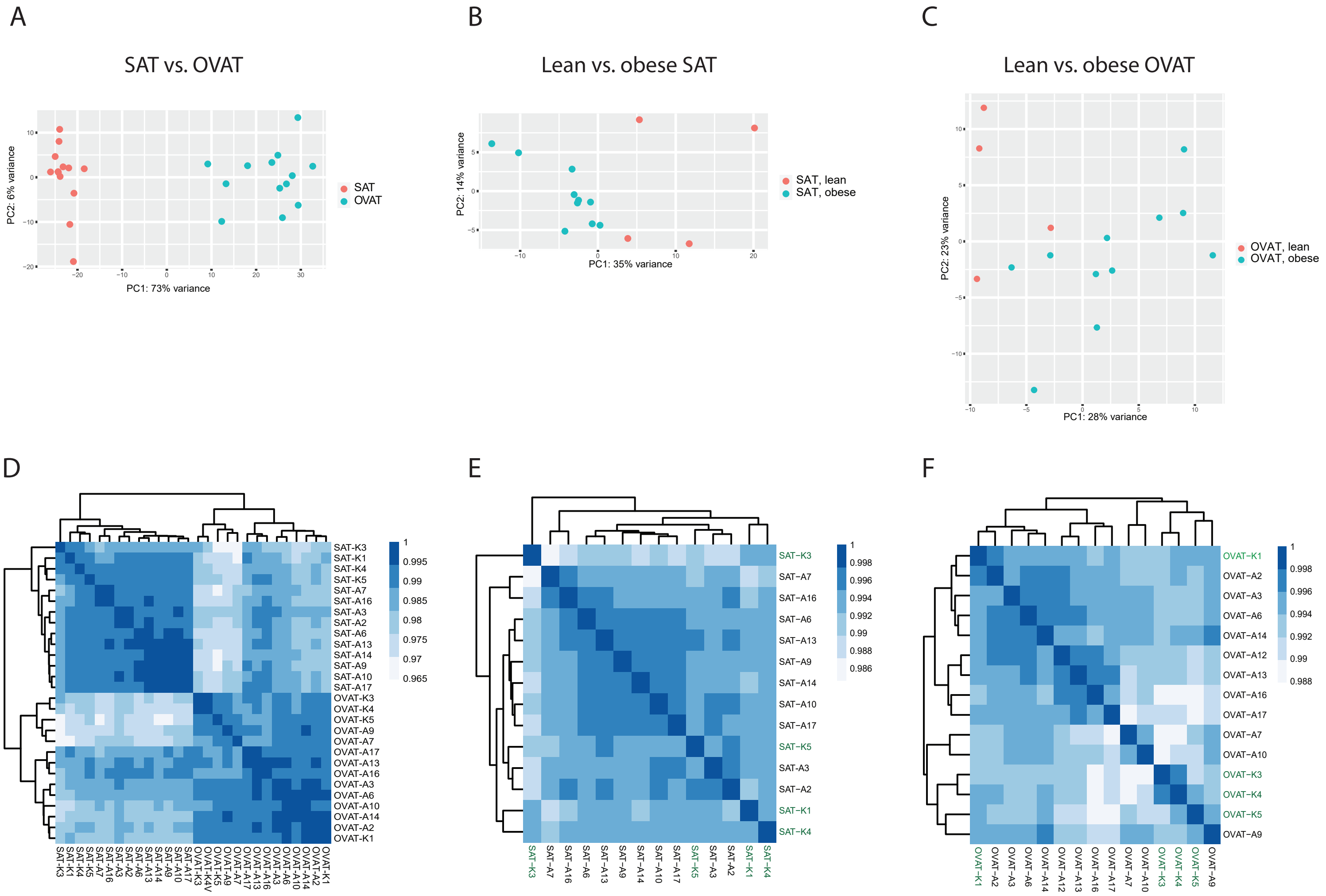

A
SAT vs. OVAT
PC1: 73% variance
PC2: 6% variance
SAT
OVAT
B
Lean vs. obese SAT
PC1: 35% variance
PC2: 14% variance
SAT, lean
SAT, obese
C
Lean vs. obese OVAT
PC1: 28% variance
PC2: 23% variance
OVAT, lean
OVAT, obese
D
E
F

Figure S5

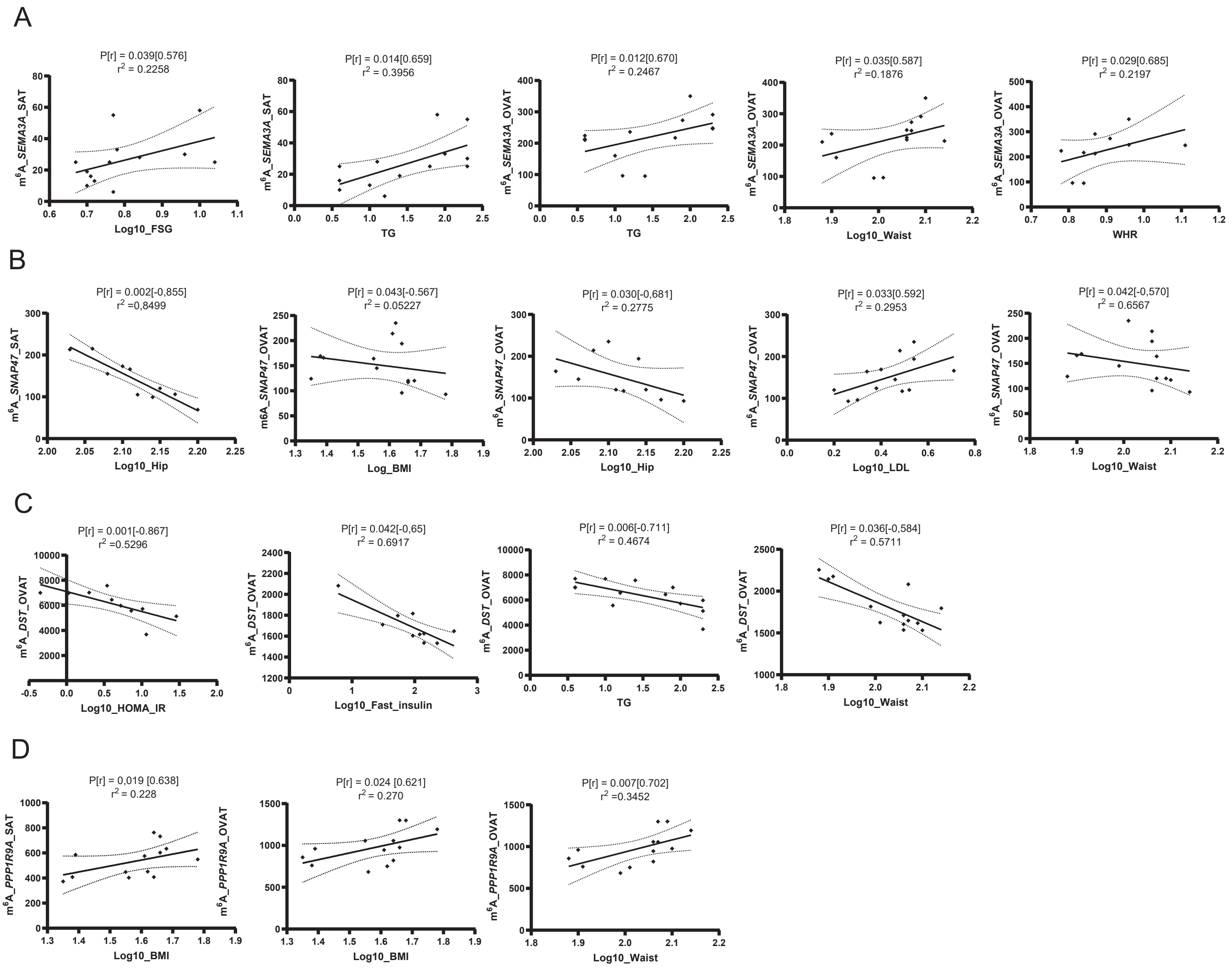

A
P[r] = 0.039[0.576]
r2 = 0.2258
m6A_SEMA3A_SAT
Log10_FSG
P[r] = 0.014[0.659]
r2 = 0.3956
m6A_SEMA3A_SAT
TG
P[r] = 0.012[0.670]
r2 = 0.2467
m6A_SEMA3A_OVAT
TG
P[r] = 0.035[0.587]
r2 =0.1876
m6A_SEMA3A_OVAT
Log10_Waist
P[r] = 0.029[0.685]
r2 = 0.2197
m6A_SEMA3A_OVAT
WHR
B
P[r] = 0.002[-0,855]
r2 =0,8499
m6A_SNAP47_SAT
Log10_Hip
P[r] = 0.043[-0.567]
r2 = 0.05227
m6A_SNAP47_OVAT
Log_BMI
P[r] = 0.030[-0,681]
r2 = 0.2775
m6A_SNAP47_OVAT
Log10_Hip
P[r] = 0.033[0.592]
r2 = 0.2953
m6A_SNAP47_OVAT
Log10_LDL
P[r] = 0.042[-0,570]
r2 = 0.6567
m6A_SNAP47_OVAT
Log10_Waist
C
P[r] = 0.001[-0.867]
r2 =0.5296
m6A_DST_OVAT
Log10_HOMA_IR
P[r] = 0.042[-0,65]
r2 = 0.6917
m6A_DST_OVAT
Log10_Fast_insulin
P[r] = 0.006[-0.711]
r2 = 0.4674
m6A_DST_OVAT
TG
P[r] = 0.036[-0,584]
r2 = 0.5711
m6A_DST_OVAT
Log10_Waist
D
P[r] = 0,019 [0.638]
r2 = 0.228
m6A_PPP1R9A_SAT
Log10_BMI
P[r] = 0.024 [0.621]
r2 = 0.270
m6A_PPP1R9A_OVAT
Log10_BMI
P[r] = 0.007[0.702]
r2 =0.3452
m6A_PPP1R9A_OVAT
Log10_Waist

Figure S6

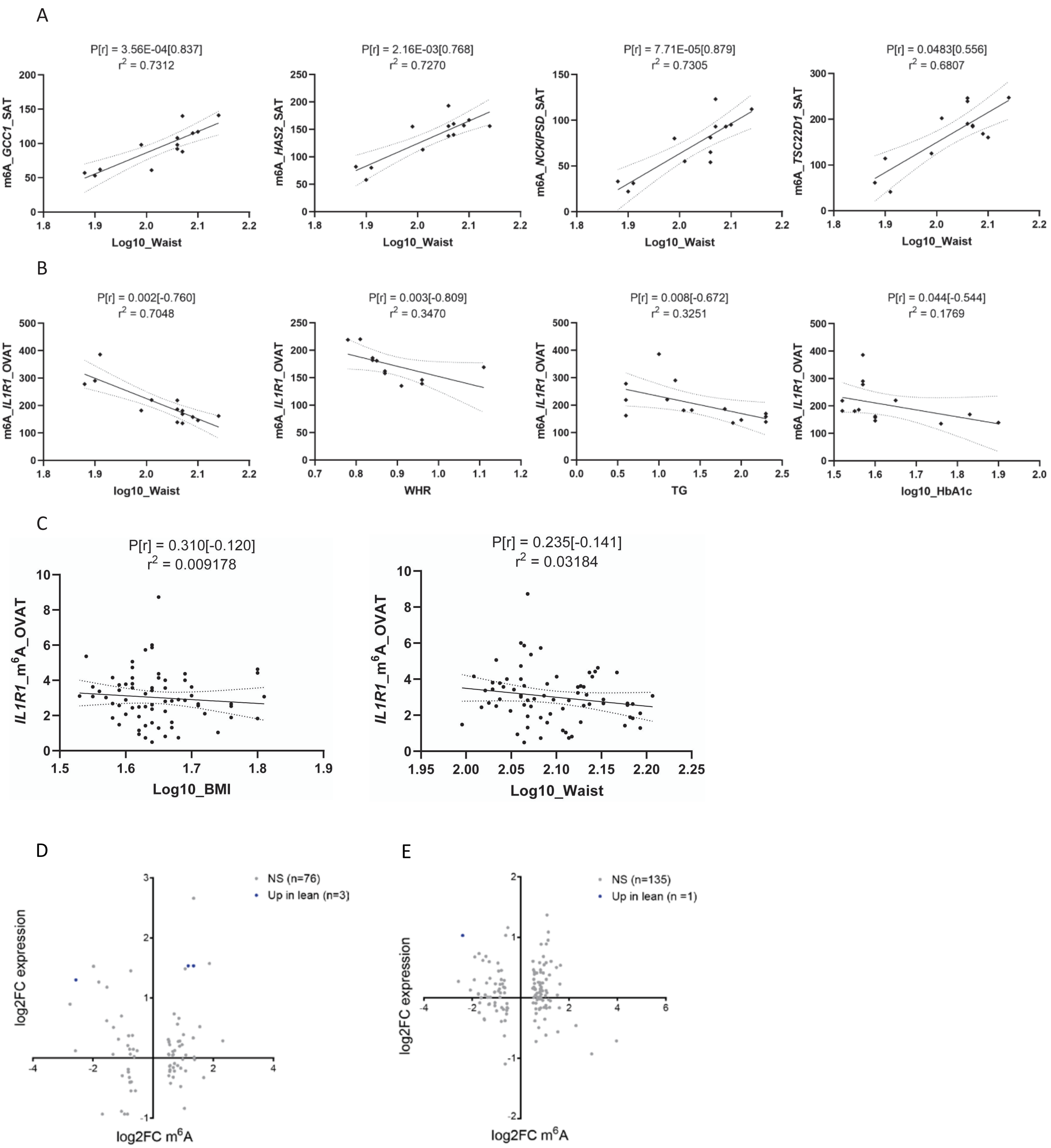
A
P[r] = 3.56E-04[0.837]
r2 = 0.7312
m6A_GCC1_SAT
Log10_Waist
P[r] = 2.16E-03[0.768]
r2 = 0.7270
m6A_HAS2_SAT
Log10_Waist
P[r] = 7.71E-05[0.879]
r2 = 0.7305
m6A_NCKIPSD_SAT
Log10_Waist
P[r] = 0.0483[0.556]
r2 = 0.6807
m6A_TSC22D1_SAT
Log10_Waist
B
P[r] = 0.002[-0.760]
r2 = 0.7048
m6A_IL1R1_OVAT
log10_Waist
P[r] = 0.003[-0.809]
r2 = 0.3470
m6A_IL1R1_OVAT
WHR
P[r] = 0.008[-0.672]
r2 = 0.3251
m6A_IL1R1_OVAT
TG
P[r] = 0.044[-0.544]
r2 = 0.1769
m6A_IL1R1_OVAT
log10_HbA1c
C
P[r] = 0.310[-0.120]
r2 = 0.009178
IL1R1_m6A_OVAT
Log10_BMI
P[r] = 0.235[-0.141]
r2 = 0.03184
IL1R1_m6A_OVAT
Log10_Waist
D
NS (n=76)
Up in lean (n=3)
log2FC expression
log2FC m6A
E
NS (n=135)
Up in lean (n =1)
log2FC expression
log2FC m6A

Figure S7

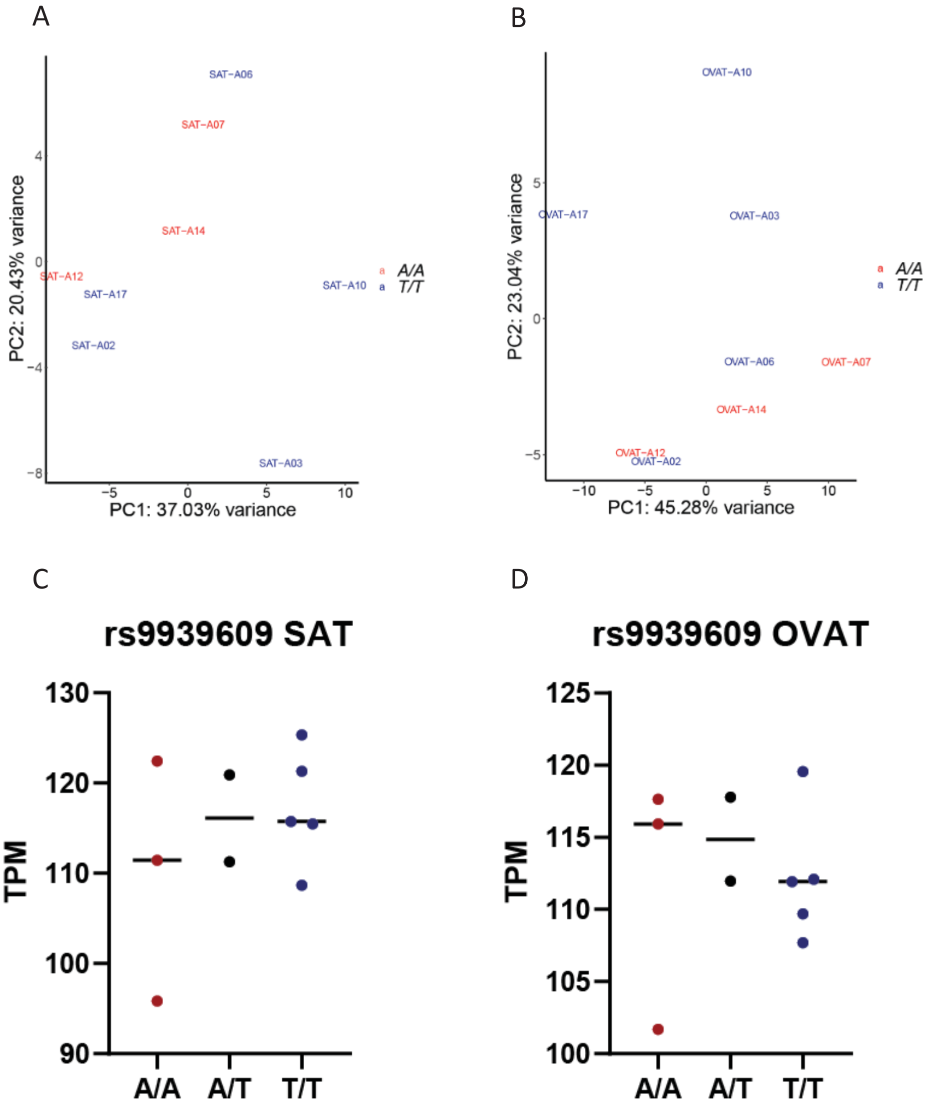
A
B
C
D
PC1: 37.03% variance
PC2: 20.43% variance
SAT−A06
SAT−A07
SAT−A14
SAT−A12
SAT−A17
SAT−A02
SAT−A10
SAT−A03
A/A
T/T
PC1: 45.28% variance
PC2: 23.04% variance
OVAT−A10
OVAT−A17
OVAT−A03
OVAT−A06
OVAT−A07
OVAT−A14
OVAT−A12
OVAT−A02
rs9939609 SAT
rs9939609 OVAT
TPM
A/A
A/T
T/T
