# Supplementary material for: Global mapping of RNA N6-methyladenosine (m6A) in human subcutaneous and visceral adipose tissue reveals novel targets that correlate with clinical variables of obesity
Source: Biomark Res. 2025 Nov 12;13:146. doi: 10.1186/s40364-025-00857-0 (PMC12613671; doi:10.1186/s40364-025-00857-0)
Supplement: Supplementary file 3 — Supplementary Material 3 [file 40364_2025_857_MOESM3_ESM.docx]

## Supplementary figure legends

**Fig. S1: Visualization of selected regions positive and negative for m^6^A in human adipose tissue.** Strand specific IGV profiles of m^6^A IP and corresponding input samples in adipogenic genes from SAT and OVAT of 3 individuals with obesity. «SAT-VAT conserved» denote peaks overlapping between all adipose tissue samples (n=26 (13 individuals, SAT, OVAT)). *GAPDH* is included as a negative control, showing no m^6^A enrichment.

**Fig. S2: Quality control of m^6^A peak data:** Enrichment of RRACH motifs and metagene profiles in peak summit regions for: A: adipose tissue (AT), B: adipocytes (AC). Motif analysis was performed with MEME, adjusted p-value is shown.

**Fig S3: m^6^A dot blot results:** 5 intra-individually paired samples of SAT and OVAT from individuals with obesity. Results show anti-m^6^A immunoblots and methylene blue as loading control.

**Fig. S4:** **Differential gene expression analysis by RNAseq.** A-C: PCA analysis and D-F: hierarchical clustering for A, D: SAT vs. OVAT (n=13), B, E: Lean vs. obese in SAT (n=3/10), C, F: Lean vs. obese in OVAT (n=3/11). Differential expression was defined as │Log2FC│ >1 and adjusted p-value < 0.01.

**Figure S5:** **Significant correlations of depot specific DMRs (SAT vs. OVAT) with clinical parameters.** m^6^A levels in DMRs of **A:** *SEMA3A*: chr7;84194552-84194851 (-), **B:** *SNAP47*; chr1:227732576-227732775 (+), **C:** *DST*; chr6:56540823-56541121 (-) **D:** *PPP1R9A;* chr7:94911116-94911414 (+). P-value [Spearmans’ rho] and r^2^ for the regression line is shown.

**Fig S6:** **Correlations and meRIP-qPCR for obesity specific DMRs (normal weight vs. obesity).** Significant correlation of m^6^A level with clinical traits in discovery cohort in selected DMRs in **A:** SAT: *GCC1*; chr7: 127585149-127585248(-), *HAS2*; chr8: 121614211-121614310(-), *TSC22D1;* chr13: 44576257-44576356(-), *NCKIPSD;* chr13: 44576257-44576356(-) and **B:** OVAT: *IL1R1*; chr2: 102176084-102176183(+).**C:** Correlations of m^6^A levels measured in *IL1R1* DMR region with BMI and waist circumference in validation cohort (meRIP-qPCR, n=72). P-value [Spearmans’ rho] and r^2^ for the regression line is shown. **D, E:** Scatter plot of expression status (log2FC determined by RNAseq) of genes with differentially methylated regions (|log2FC| ≥ 1.5, FDR < 0.1) between lean and obese in **E:** SAT and **F:** OVAT.

**Figure S7:** **Differential m^6^A and gene expression analysis in *FTO* risk allele rs9939609 carriers. A, B:** PCA plot of global methylation level in **A:** SAT and **B:** OVAT, generated by RADAR, subjects grouped by genotype (individuals with obesity homozygous for risk or wild type variant). **C, D:** Expression of *FTO* (RNA-seq data, transcript per million (TPM)) grouped on rs9939609 genotype in **C:** SAT, **D:** OVAT. rs9939609 risk allele: A, wild-type allele: T.
